# Visible-Light-Driven Co_3_O_4_/Nb_2_O_5_ Heterojunction Nanocomposites for Efficient Photocatalytic and Antimicrobial Performance in Wastewater Treatment

**DOI:** 10.3390/molecules30122561

**Published:** 2025-06-12

**Authors:** Anil Pandey, Santu Shrestha, Rupesh Kandel, Narayan Gyawali, Subas Acharya, Pujan Nepal, Binod Gaire, Vince Fualo, Jae Ryang Hahn

**Affiliations:** 1 Department of Chemistry, Jeonbuk National University, Jeonju 54896, Republic of Korea; pandeyan2001@gmail.com (A.P.); santu_shrestha@hotmail.com (S.S.); naran.gywli@gmail.com (N.G.); link2subas@gmail.com (S.A.); pujannepal25@gmail.com (P.N.); gairebinod@gmail.com (B.G.); fualo.vc@gmail.com (V.F.); 2 Department of Bionanotechnology and Bioconvergence Engineering, Graduate School, Jeonbuk National University, Jeonju 54896, Republic of Korea; rupkandel87@gmail.com; 3 Textile Engineering, Chemistry and Science, North Carolina State University, 2401 Research Dr., Raleigh, NC 27695-8301, USA

**Keywords:** heterojunction interface, Co_3_O_4_/Nb_2_O_5_ visible-light-driven photocatalyst, synergistic effect, water pollutants, antibacterial activities

## Abstract

The development of high-performance photocatalysts is vital for combating water pollution and microbial contamination. In this study, visible-light-active Z-scheme heterojunction nanocomposites composed of Co_3_O_4_ and Nb_2_O_5_ (CNNC) were synthesized via co-crystallization and subsequent high-pressure annealing to enhance photocatalytic and antimicrobial performance. Structural and optical analyses via XRD, FESEM, TEM, XPS, and PL confirmed the heterojunction formation between porous Co_3_O_4_ nanoparticles (CONP) and columnar orthorhombic Nb_2_O_5_ nanoparticles (NONP). The CNNC exhibited significantly improved photocatalytic activity, achieving degradation efficiencies of 95.1% for methylene blue, 72.6% for tetracycline, and 90.0% for Congo red within 150 min. Kinetic studies showed that CNNC’s rate constants were 367% and 466% of those of CONP and NONP, respectively. Moreover, CNNC demonstrated a strong antibacterial effect on *Staphylococcus aureus* and *Escherichia coli* with ZOI values of 9.3 mm and 6.8 mm, respectively. Mechanistic analysis revealed that the Z-scheme charge-transfer pathway improved charge separation and reduced electron–hole recombination, contributing to the promoted photocatalytic efficiency. The nanocomposite also showed robust stability and recyclability over five times. These results highlight the promise of CNNC as a bifunctional, visible-light-driven photocatalyst for pollutant decomposition and microbial control.

## 1. Introduction

Infective bacteria are responsible for a wide range of infectious diseases, employing sophisticated virulence mechanisms to invade host cells, evade immune responses, and ensure their survival and proliferation [1,2]. Among these mechanisms, the secretion of toxins that target and damage immune cells plays a crucial role in undermining host defenses. Additionally, bacteria frequently form biofilms—organized colonies of microorganisms enclosed in a self-produced extracellular matrix—that confer resistance to immune clearance and antibiotic treatment while facilitating nutrient acquisition [3]. The appearance and rapid spread of antibiotic-resistant strains have rendered many conventional therapies ineffective, highlighting an urgent need for alternative antibacterial strategies. Recent advances in nanotechnology have opened new avenues for antimicrobial intervention. Nanoscale metal oxide materials and their composites have emerged as potent antibacterial agents, exhibiting intrinsic antimicrobial properties and the ability to bypass conventional resistance mechanisms through direct interactions with bacterial cell walls, leading to membrane disruption and cell death [4,5]. This non-traditional approach offers promising prospects for addressing multidrug-resistant bacterial infections.

Concurrently, industrialization and urban development have significantly exacerbated environmental pollution, with organic contaminants posing severe risks to ecosystems and public health [6,7]. Water pollution, in particular, remains one of the most urgent global environmental concerns [8]. The discharge of untreated dyes and pharmaceutical compounds—like methylene blue (MB), methyl orange (MO), Congo red (CR), and tetracycline (TC)—into aquatic systems has led to persistent contamination due to their intricate molecular architectures and resilience to natural degradation processes [9,10,11,12,13]. The coexistence of these pollutants with pathogenic microbes in water sources necessitates integrated solutions that can simultaneously address both chemical and biological contamination. Advanced oxidation processes, especially semiconductor-based photocatalysis, have garnered significant awareness for environmental remediation due to their capability to harness solar energy to decompose organic pollutants and inactivate microbes [14,15,16]. Since the foundational work by Fujishima and Honda on TiO_2_ [17], various semiconductors, including ZnO, CdS, SrTiO_3_, BiOI, Co_3_O_4_, and Nb_2_O_5_, have been studied for photocatalytic applications. Photocatalysis operates by generating reactive oxygen species (ROS) through photogenerated charge carrier pairs, effectively decomposing pollutants and disrupting microbial integrity [18,19,20,21]. Compared to the established methods like adsorption, membrane filtration, or ozonation, photocatalysis offers advantages, including cost-effectiveness, sustainability, and easy catalyst recovery [20,22,23,24].

However, the performance of photocatalytic systems largely relies on the optical features and charge-transfer dynamics of the semiconductor. Nb_2_O_5_ is a promising *n*-type semiconductor with excellent chemical stability (~700–800 °C) [25], low toxicity [26], and suitable redox potential (E_CB_ = −0.52 eV, E_VB_ = +2.61 eV) [27]. Despite these merits, its functional applicability is limited by a band gap (3.1–3.4 eV), poor visible (VL) light absorption, and rapid recombination of charge carriers [28,29,30,31,32]. Various strategies, including doping, morphology tuning, and heterojunction construction, have been explored to overcome these limitations [29,33,34]. Among these, coupling Nb_2_O_5_ with other semiconductors to create heterojunctions—especially Z-scheme configurations—offers an efficient pathway to enhance VL responsiveness, promote charge separation, and optimize photocatalytic efficiency. Tian et al. reported the optimum yield of photocatalytic CH_4_ and CO because of the directional segregation of charged species through the Z-scheme heterojunction of CdS/Nb_2_O_5_ [35]. Aimaiti et al. reported the highly efficient photocatalytic removal of RhB and Cr(VI) using the S-scheme heterojunction of Nb_2_O_5_/red phosphorus [30]. Wang et al. reported the enhanced efficiency for hydrogen evolution and 5-hydroxymethylfurfural oxidation due to the prolonged lifetime of the charge carriers through a Z-scheme ZnIn_2_S_4_/Nb_2_O_5_ heterojunction [36]. However, there are limited studies on the antibacterial applications of Nb_2_O_5_. A few studies have been reported on Nb_2_O_5_ as a bioactive and reinforcing additives in dental materials. Particularly, the incorporation of Nb_2_O_5_ has been shown to enhance the antibacterial activity of other materials, such as NF_TiO_2_ nanoparticles and CaF_2_–CaO–B_2_O_3_–P_2_O_5_–SrO glass [37,38]. Safavi et al. have also demonstrated significantly improved antibacterial efficacy with the addition of Nb_2_O_5_ to hydroxyapatite (HAp) [39].

Co_3_O_4_, a *p*-type semiconductor with a variable band gap (1.2–5 eV), exhibits superior VL absorption and unique electronic features owing to its spinel structure, which includes Co^2+^ and Co^3+^ ions in tetrahedral and octahedral sites, respectively [40,41]. Although Co_3_O_4_ shows potential as a VL-active photocatalyst, its standalone performance is hindered by rapid electron–hole recombination. Nevertheless, it has proven to be an excellent component for constructing *p*-*n* or Z-scheme heterojunctions with *n*-type semiconductors, resulting in enhanced photocatalytic activity through synergistic band alignment and improved charge carrier dynamics [42,43]. Noteworthy examples include Co_3_O_4_-based composites with ZnO, TiO_2_, CeO_2_, and WO_3_ for photocatalytic degradation of organic pollutants [42,44,45,46,47]. In addition, Co_3_O_4_-based composites are also reported for their antibacterial activities. Co/Co_3_O_4_ is reported for its vigorous antibacterial activity against *Pseudomonas aeruginosa* and decolorization of organic dyes [48]. Co_3_O_4_/BiOCl has been reported for its significant antibacterial activity due to the synergistic effect of mesoporous structure, photothermal effect, and ROS production [49]. CoO_x_/MnO_x_ incorporated GO is reported for its effective performance in MB removal and antibacterial activity [50]. However, to date, no studies have reported the combination of Co_3_O_4_ with Nb_2_O_5_ in a heterojunction photocatalyst.

Therefore, this study reports a novel nanocomposite of Co_3_O_4_/Nb_2_O_5_ (CNNC) synthesized through co-crystallization and subsequent annealing at high temperature and high pressure for its photocatalytic and antibacterial applications for the first time. Given the complementary properties of Nb_2_O_5_ (strong oxidation ability) and Co_3_O_4_ (VL sensitivity), constructing a Co_3_O_4_/Nb_2_O_5_ (CNNC) heterojunction is expected to yield a Z-scheme photocatalyst with superior performance for both pollutant degradation and microbial disinfection. Such a configuration facilitates directional charge transfer, minimizes charge recombination, and expands light absorption, thereby enhancing overall photocatalytic efficiency. The wide band gap of Nb_2_O_5_ makes it an excellent candidate for bandgap engineering when coupled with Co_3_O_4_, facilitating favorable band-edge alignment and enabling the efficient generation of reactive oxygen species (ROS) such as O_2_^−^• and •OH. The uniform distribution of porous cubic-phase Co_3_O_4_ over irregularly shaped orthorhombic Nb_2_O_5_ results in a high-surface-area nanocomposite with abundant active sites. This morphology enhances light harvesting, promotes pollutant adsorption, and increases contact with bacterial cells. Furthermore, the rough surface of the nanocomposite contributes to antibacterial efficacy through physical disruption of bacterial membranes. Collectively, these factors account for the superior photocatalytic and antibacterial performance of the CNNC nanocomposites. Moreover, Z-scheme heterostructures have demonstrated greater redox capability and stability than conventional type I or type II heterojunctions.

In the current work, we describe the preparation of Co_3_O_4_/Nb_2_O_5_ nanocomposites via a simple, one-step, capping-agent-free co-crystallization method followed by high-pressure annealing. This green and scalable approach yields structurally integrated composites with improved interfacial contact and crystallinity. The optimized CNNC-1 (1:1 Co_3_O_4_:Nb_2_O_5_) exhibited remarkable photocatalytic activity, achieving 95.1% degradation of methylene blue, 90.0% of Congo red, and 72.6% of tetracycline on VL exposure. The corresponding rate constant was ~4-fold and ~5-fold higher than those of pristine Co_3_O_4_ and Nb_2_O_5_, respectively. Band structure analysis and scavenger studies supported a Z-scheme charge-transfer process. Furthermore, the CNNC-1 exhibited notable antibacterial performance against *Escherichia coli* and *Staphylococcus aureus*, as well as good reusability over multiple cycles. This work introduces a novel bifunctional nanocomposite photocatalyst with dual capabilities for environmental detoxification and antimicrobial action, addressing critical challenges in water purification. The proposed CNNC platform offers a promising direction for developing multifunctional, visible-light-driven photocatalysts for real-world applications.

## 2. Results and Discussion

### 2.1. Surface Morphology and Elemental Analysis

**Figure 1:**
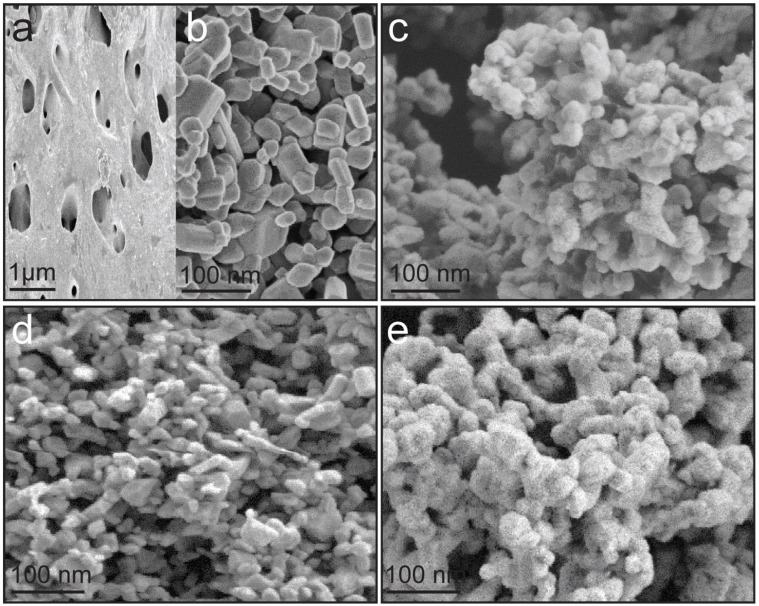
FE-SEM images of nanoparticles and nanocomposites: (**a**) porous CONP, (**b**) columnar-shaped NONP, (**c**) CNNC-1 (1:1), (**d**) CNNC-2 (1:2), and (**e**) CNNC-3 (2:1).

The morphological characteristics of CONP, NONP, and CNNC nanocomposites—CNNC-1 (1:1), CNNC-2 (1:2), and CNNC-3 (2:1)—were examined using FE-SEM, as displayed in Figure 1. Figure 1a reveals that CONP exhibits a porous bulk structure with a relatively smooth surface, consistent with previous reports [51,52]. In contrast, Figure 1b shows that NONP nanoparticles possess an irregular shape with a dominant columnar orthorhombic morphology, aligning with earlier observations [30,31]. The FE-SEM images of CNNC-1, CNNC-2, and CNNC-3 (Figure 1c-e) demonstrate the successful formation of nanocomposites, where CONP nanoparticles are uniformly distributed across the columnar-shaped NONP surfaces. The particle size distribution of the CNNC nanocomposites was investigated using the ImageJ software (ImageJ 1.53e) and Gaussian curve fitting (OriginPro 2016), and the histogram is presented in Figure S1 and tabulated (Table S1). The average particle size of the composites is 95.1, 72.5, and 105.0 nm for CNNC-1, CNNC-2, and CNNC-3, respectively. The particle size decreased with increasing ratio of Nb_2_O_5_, which might be due to more nucleation sites provided by Nb_2_O_5_.

While no significant morphological differences were observed among the three composites, CNNC-3 (Figure 1e) displayed a noticeably rougher surface, likely due to its higher CONP content. However, distinguishing the individual morphologies of CONP and NONP within the CNNC-1 sample from SEM alone proved challenging. To address this limitation and achieve better structural resolution, HR-TEM was used to further investigate CNNC-1, as discussed in a later section.

Elemental composition and spatial distribution in CNNC-1 were further examined using EDS. The EDS elemental scanning (Figure S2a–e) confirmed the homogeneous presence of Co, Nb, and O throughout the composite. Additionally, the EDS spectrum (Figure S2f) validated the purity of the composite, revealing the presence of only Co (11.8%), Nb (11.1%), and O (77.2%), with no detectable impurities. These results confirm that CNNC-1 consists exclusively of cobalt and niobium oxides.

**Figure 2:**
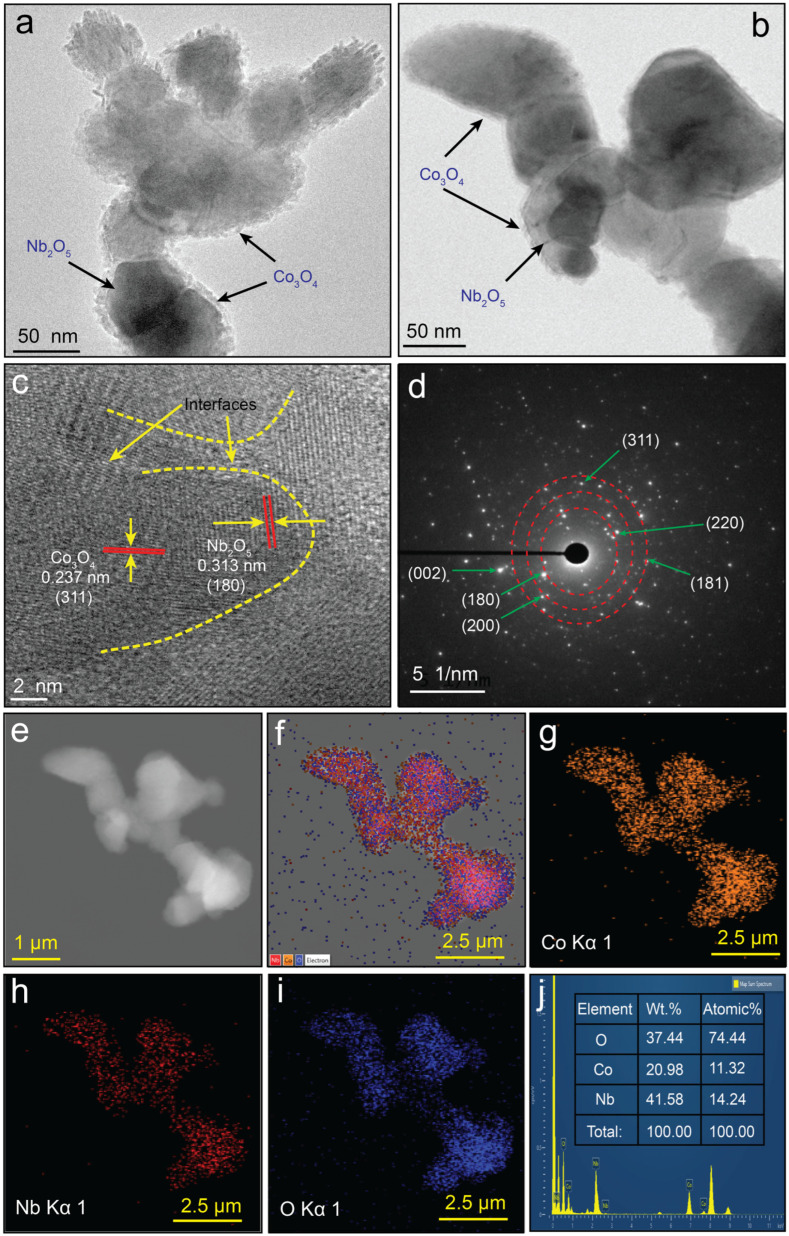
(**a**,**b**) HR-TEM images; (**c**) lattice fringes of CONP and NONP at the interface; (**d**) SAED pattern; (**e**) EDS image; (**f**–**i**) EDS elemental mappings; and (**j**) the EDS spectrum of CNNC-1.

To gain an in-depth understanding of the morphological characteristics of CNNC-1, HR-TEM was conducted, as depicted in Figure 2a–d. The low-magnification TEM images (Figure 2a,b) reveal the presence of two distinct nanostructured phases in close contact. A dense, darker region corresponds to NONP, consistent with the columnar morphology observed in the FE-SEM micrograph (Figure 1b), while a lighter, porous region is attributed to agglomerated CONP. These porous Co_3_O_4_ particles are uniformly distributed over the surface of Nb_2_O_5_ and also appear as agglomerates within the composite matrix. The higher proportion of CONP compared to NONP in the composite can be ascribed to their smaller particle size and high porosity, as indicated by BET (surface area) and BJH (pore volume) analyses (Table S2).

This high porosity is expected to considerably promote the photocatalytic performance of the composite by increasing its active surface area and facilitating pollutant adsorption and reactive species generation. Figure 2c displays lattice fringes with interplanar distances of 0.313 nm and 0.237 nm, corresponding to the (180) facet of orthorhombic Nb_2_O_5_ (JCPDS 30-0873) and the (311) plane of cubic spinel Co_3_O_4_ (JCPDS 00-042-1467), respectively [27,53]. The simultaneous observation of these lattice fringes at the interface confirms the coexistence of both components and strongly supports the formation of a heterojunction in CNNC-1.

Such a heterojunction structure would facilitate efficient interfacial charge transfer between Co_3_O_4_ and Nb_2_O_5_, thereby suppressing the recombination of photoinduced charge carriers and enhancing photocatalytic efficiency. Furthermore, the SAED pattern (Figure 2d) discloses distinct diffraction rings, confirming the polycrystalline nature of the CNNC-1 composite. The observed diffraction planes are consistent with the XRD results discussed in Section 2.2, further validating the successful formation of the Co_3_O_4_/Nb_2_O_5_ heterojunction.

### 2.2. Structural Characterization

**Figure 3:**
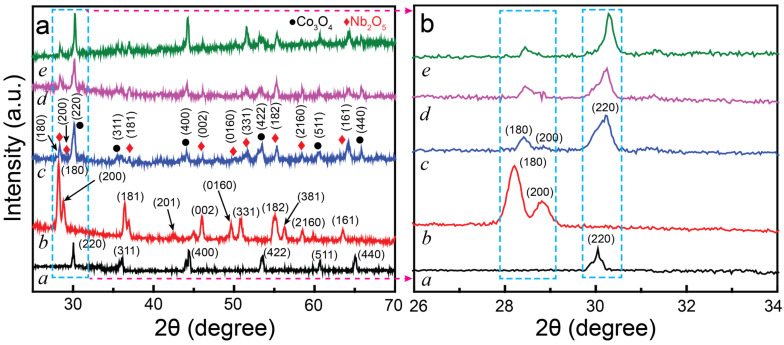
(**a**) HR-XRD profiles and (**b**) magnified XRD profiles in the range of 26° to 34° for (*a*) CONP, (*b*) NONP, (*c*) CNNC-1, (*d*) CNNC-2, and (*e*) CNNC-3.

The crystal structure and phase purity of CONP, NONP, and the CNNC nanocomposites (CNNC-1, CNNC-2, and CNNC-3) were examined via high-resolution XRD, as shown in Figure 3a,b. In Figure 3a, the diffraction pattern of CONP (spectrum *a*) displays well-defined peaks characteristic of a spinel Co_3_O_4_ nanosheet structure. Prominent peaks appear at 2*θ* values of 30.14°, 34.72°, 42.80°, 50.22°, 52.13°, and 63.39°, corresponding to the (220), (311), (400), (422), (511), and (440) crystal facets, respectively, which match the cubic phase of Co_3_O_4_ (JCPDS 00-042-1467) [54]. Similarly, the XRD pattern of NONP (spectrum *b*) shows distinct diffraction signals at 28.22°, 28.75°, 36.42°, 42.44°, 45.99°, 49.62°, 50.79°, 55.10°, 56.22°, 58.45°, and 63.51°. These peaks correspond to the (180), (200), (181), (201), (002), (0160), (331), (182), (381), (2160), and (161) planes, respectively, confirming the orthorhombic phase of Nb_2_O_5_ (JCPDS No. 30-0873) [55,56]. The XRD patterns of the CNNC nanocomposites (Figure 3a, curves c–e) exhibit diffraction peaks attributed to both Co_3_O_4_ and Nb_2_O_5_, showing the successful composite formation. No additional peaks were found, confirming the high purity of the synthesized samples and the unavailability of secondary phases.

The average crystallite sizes of CNNC-1, CNNC-2, and CNNC-3 were determined using the following Scherrer’s equation:

*D* = *kλ*/(*β* cos*θ*), (1)

where *D* is the crystallite size; *k* indicates the shape factor; *λ* represents the X-ray wavelength; *β* denotes the full width at half maximum (FWHM) of the most intense peak, and *θ* is the Bragg angle. Based on the Gaussian fitting of the (220) diffraction peak, the average crystallite sizes were determined to be 17.45 nm for CNNC-1, 19.05 nm for CNNC-2, and 20.68 nm for CNNC-3. For comparison, the crystallite size calculated using the Williamson–Hall plot is presented in Table S3. The structural parameters, such as crystallinity, lattice constant, and dissociation density of the materials, are also presented in it. These results suggest that the crystallite size remains nearly constant across the heterojunction samples, indicating structural stability upon composite formation.

Figure 3b presents an enlarged view of the XRD patterns, highlighting the relative intensities of key peaks. The (200) peak associated with Co_3_O_4_ exhibits higher intensity than the (180) peak of Nb_2_O_5_, particularly in composites with higher Co_3_O_4_ content. This enhanced intensity may be attributed to the larger volume fraction of Co_3_O_4_, likely due to its smaller crystallite size and higher surface area contribution in the composite.

**Figure 4:**
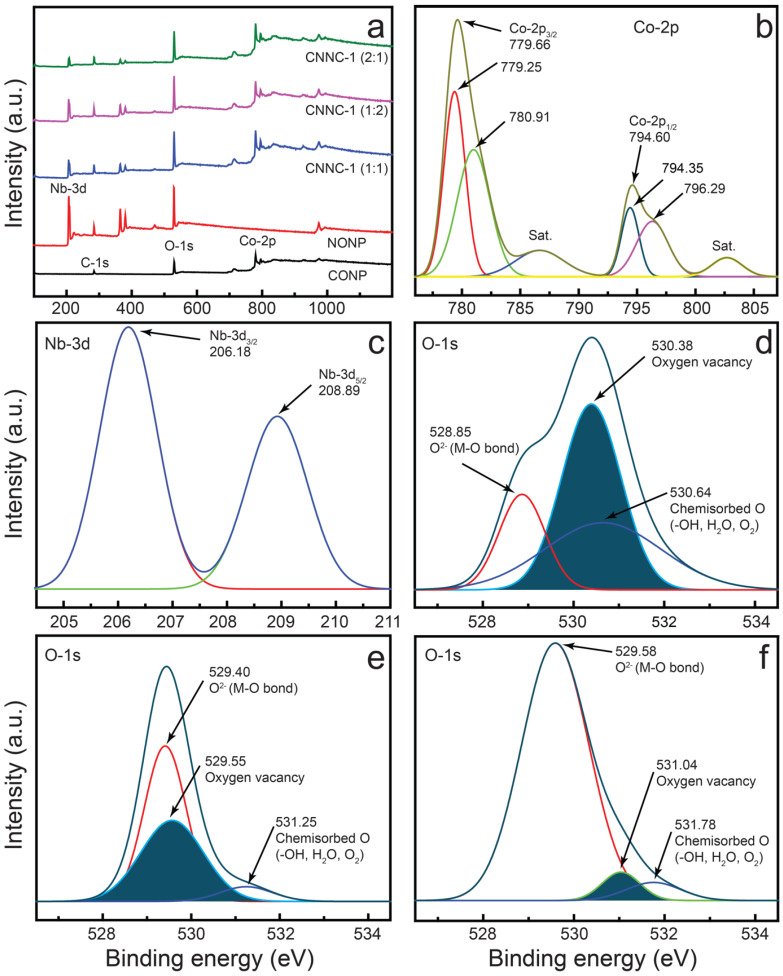
(**a**) XPS survey spectra of nanoparticles and nanocomposites; (**b**) Co 2p spectrum; (**c**) Nb 3d spectrum of the CNNC-1 composite; and (**d**–**f**) O 1s spectra of CONP, NONP, and CNNC-1.

To examine the surface composition and chemical states of CONP, NONP, and CNNC samples, high-performance XPS was employed. The survey profile (Figure 4a) confirms the availability of key elements in each sample: cobalt and oxygen in CONP; niobium and oxygen in NONP; and all three elements—Co, Nb, and O—in the CNNC composite, indicating successful integration of both metal oxides.

The high-resolution Co-2p spectrum of CNNC-1 is presented in Figure 4b. The Co-2p region can be deconvoluted into four distinct peaks. Peaks at 779.66 eV and 794.60 eV correspond to Co-2p_3/2_ and Co-2p₁_/2_, respectively, with an energy separation of approximately 15.0 eV, along with shake-up satellites observed at 786.56 eV and 802.0 eV [57]. Additionally, peaks at 779.25 eV and 794.35 eV are attributed to Co^3+^, while those at 780.91 eV and 796.29 eV correspond to Co^2+^, indicating the co-presence of both Co(II) and Co(III) oxidation states in the composite. A similar Co-2p profile was observed in CONP (Figure S3a), albeit with slightly higher binding energies.

The Nb-3d spectrum of CNNC-1 (Figure 4c) shows two separate signals at 206.18 eV and 208.89 eV, assigned to Nb-3d_5/2_ and Nb-3d_3/2_, respectively, which is consistent with the Nb^5+^ oxidation state [58]. These peaks also appear in NONP, again with marginally higher binding energies (Figure S3b). The observed shifts in the binding energies of both Co-2p and Nb-3d in the composite relative to the pristine materials suggest strong electronic interactions and the formation of chemical bonds between Co_3_O_4_ and Nb_2_O_5_, indicative of heterojunction formation.

Figure 4d–f shows the deconvoluted O-1s spectra of CONP, NONP, and CNNC-1, respectively. In CONP (Figure 4d), peaks at 528.85 eV, 530.38 eV, and 530.64 eV are related to lattice oxygen, oxygen vacancies, and chemisorbed oxygen species [59]. For NONP (Figure 4e), signals at 529.40 eV, 529.55 eV, and 531.25 eV correspond to Nb–O bonds, oxygen vacancies, and surface oxygen species. The O-1s spectrum of CNNC-1 (Figure 4f) shows three peaks at 529.58 eV, 531.04 eV, and 531.78 eV, which are ascribed to lattice oxygen (Co–O/Nb–O), oxygen vacancies, and chemisorbed species, including –OH, H_2_O, or O_2_^2−^ [8,58,59], respectively.

Notably, the composite spectrum exhibits an increased intensity of lattice oxygen and a reduced intensity of oxygen vacancy peaks compared to the individual components. This change likely reflects the altered oxygen environment resulting from the formation of a heterojunction between CONP and NONP. Such heterojunction formation is known to promote photocatalytic efficiency by promoting charge transfer and reducing recombination losses [49]. In addition, the detailed elemental spectra of CNNC-2 and CNNC-3 are provided in Figure S4a–f. The Co-2p and Nb-3d spectra obtained for CNNC-2 and CNNC-3 are similar to CNNC-1. However, an increase in the intensity of oxygen vacancy is observed in CNNC-3 (Figure S4f), indicating an increase in defect sites [49]. The increased oxygen vacancy in the CNNC-3 could be attributed to the high CONP content and synergistic interactions between NONP and CONP.

**Figure 5:**
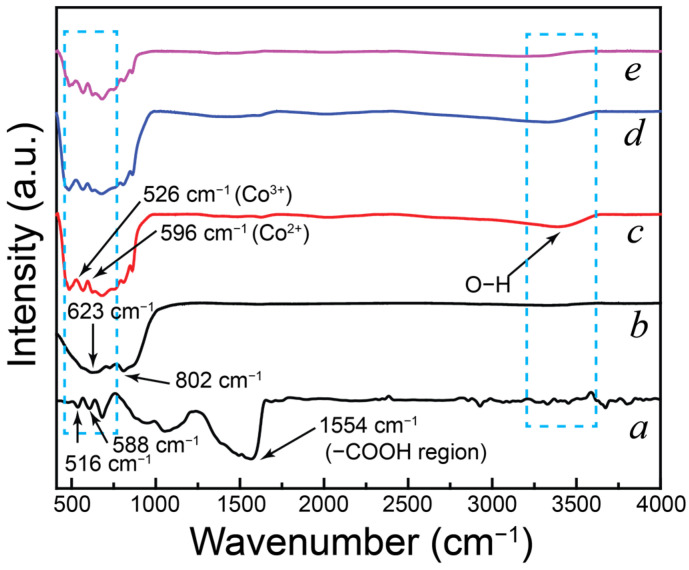
FT-IR profiles of (***a***) CONP, (***b***) NONP, (***c***) CNNC-1, (***d***) CNNC-2, and (***e***) CNNC-3.

FT-IR spectroscopy was used to investigate the chemical groups present in the materials. Figure 5 displays the FTIR spectra of CONP, NONP, and the CNNC nanocomposites (CNNC-1, CNNC-2, and CNNC-3). In the spectrum of CONP (Figure 5*a*), a strong absorption band appears at 516 cm^−1^, which corresponds to Co^3+^–O vibrations, while the band at 588 cm^−1^ is assigned to Co^2+^–O. A peak at 661 cm^−1^ is ascribed to the bridging vibrations of the O–Co–O bond [60]. Additional bands observed at 1033 cm^−1^ and 1554 cm^−1^ are associated with C–O stretching and CO_3_^2−^ anions, respectively. The broad band near 1642 cm^−1^ and 3400 cm^−1^ corresponds to molecular water bending and O–H stretching vibrations, respectively [60,61].

The FT-IR spectrum of NONP (Figure 5*b*) shows a broad band between 3200 and 3600 cm^−1^, characteristic of O–H stretching from surface hydroxyl groups. In the fingerprint region (450–900 cm^−1^), several peaks are observed, corresponding to the distinctive vibrational modes of Nb_2_O_5_ [58]. Particularly, peaks at 623 cm^−1^ and 802 cm^−1^ are related to Nb–O–Nb bridging vibrations and Nb–O stretchings, respectively [30,56].

The FT-IR spectra of the nanocomposites (Figure 5*c–e*) exhibit all major absorption bands observed in the individual components, including those corresponding to Co–O and Nb–O vibrations (450–850 cm^−1^), CO_3_^2−^ or C–O vibrations (~1554 cm^−1^), and surface-adsorbed O–H groups and water (~3400 cm^−1^). However, noticeable changes in the intensity of several bands are observed in the composite spectra, particularly in the metal–oxygen vibration region. These intensity variations suggest strong interfacial interactions between CONP and NONP, indicative of successful composite formation and chemical bonding.

### 2.3. Optical Characterization and Charge-Transfer Efficiency

The optical bandgap is a fundamental parameter that governs the electronic transitions in photocatalysts, as it determines the energy required for electrons to be excited from the valence band (VB) to the conduction band (CB). To assess the optical properties of the synthesized materials, UV–Vis spectroscopy was performed. The bandgap energies were estimated using Tauc plots following the method described by Makula et al. [62], the Tauc equation:

(*α*(*hν*)^2^) ^n^ = *A* (*hν* – *E*_g_) (2)

In this equation, *A* is a constant; *n* varies in the nature of the electronic transition (*n* = 1/2 for indirect transitions and *n =* 2 for direct transitions); *α* denotes the absorption coefficient; *h* represents Planck’s constant; *ν* is the light frequency, and *E*_g_ indicates the optical bandgap.

**Figure 6:**
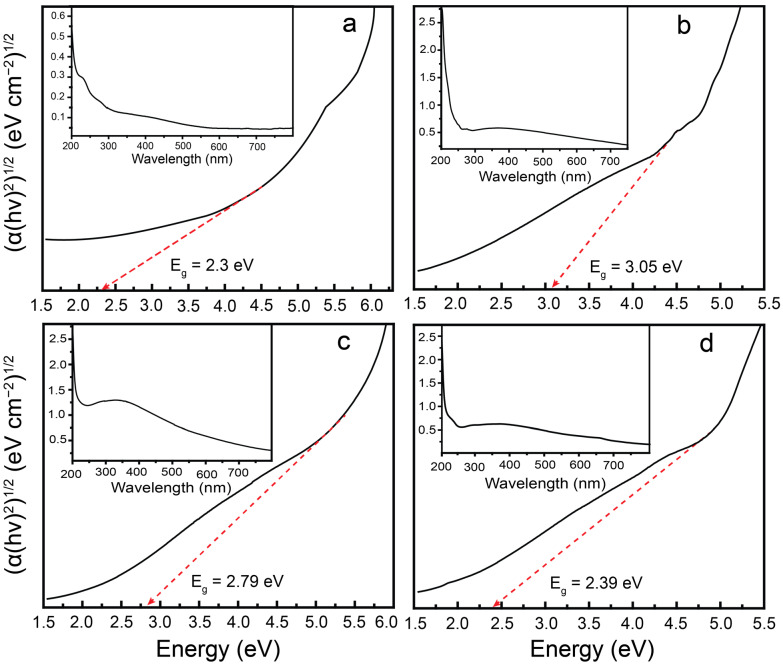
Tauc plots of (**a**) CONP, (**b**) NONP, (**c**) CNNC-2, and (**d**) CNNC-1. The insets show the corresponding UV–Vis absorbance spectra for each sample.

The UV–Vis absorbance plots of the samples are presented in the insets of Figure 6a–d and Figure S5. The corresponding Tauc plots, constructed by plotting ((*α*(*hν*)^2^)^n^ versus photon energy, were used to extrapolate the linear region and determine the optical bandgap values. The calculated bandgap energies for CONP, NONP, CNNC-1, CNNC-2, and CNNC-3 were determined to be 2.30 eV, 3.05 eV, 2.39 eV, 2.79 eV, and 2.61 eV, respectively. These results demonstrate a substantial reduction in bandgap for the CNNC composites compared to pristine NONP, indicating improved VL absorption. The bandgap values of CNNC composites lie between those of CONP and NONP, indicating strong coupling between them. The presence of the cubic spinel phase of CONP and the orthorhombic phase of NONP in the XRD spectra of nanocomposites also supports the formation of a heterojunction in CNNC composites. In addition, SEM images reveal a uniform distribution of porous CONP over irregularly shaped NONP nanoparticles, indicating a strong interfacial contact that further supports the formation of a strong heterojunction in the nanocomposites. These findings agree with previous studies on Nb_2_O_5_- and Co_3_O_4_-based composites [54,63]. The narrower bandgaps facilitate the photoexcitation of a larger number of electron–hole pairs under VL, enhancing the generation of reactive oxygen species and thereby promoting superior photocatalytic efficiency of the composites.

**Figure 7:**
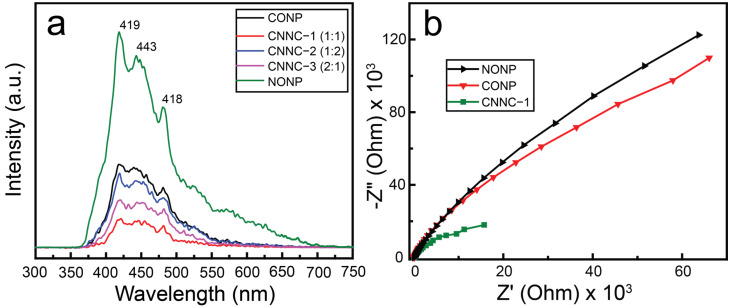
(**a**) PL spectra (λ_ex_ = 300 nm) and (**b**) EIS spectra of synthesized samples.

Photoluminescence (PL) spectroscopy was employed to examine the charge carrier recombination behavior and interfacial charge-transfer efficiency of the synthesized photocatalysts. In general, higher PL intensity corresponds to rapid recombination of photoexcited carriers, while lower PL intensity suggests more efficient electron–hole separation and transfer [64]. Figure 7a presents the PL emission spectra of CONP, NONP, and their CNNC nanocomposites, recorded under an excitation wavelength of 300 nm. Among all samples, NONP exhibited the highest PL intensity, with prominent emission peaks at approximately 419 nm, 443 nm, and 481 nm. The strong emission at 419 nm is ascribed to near-band-edge recombination of excitons, while the peaks at 443 nm and 481 nm are related to radiative recombination involving oxygen vacancies and defect-related trap states in the Nb_2_O_5_ lattice [65].

In comparison, CONP showed significantly lower PL intensity than NONP, indicating an enhanced rate of electron–hole separation. All CNNC composites exhibited a further reduction in PL intensity relative to their individual components, confirming the promoted separation of photoexcited carriers due to heterojunction formation. Notably, CNNC-1 demonstrated the lowest intensity among the composites, indicating the most efficient suppression of recombination due to optimized charge transfer at the Co_3_O_4_/Nb_2_O_5_ interface. This improved charge separation in CNNC-1 is expected to significantly contribute to its superior photocatalytic performance by facilitating the availability of charge carriers for redox reactions [65].

The charge pair separation efficiency of the CNNC-1 and pure samples was further studied via electrochemical impedance spectroscopy (EIS). The Nyquist plots obtained are presented in Figure 7b. The EIS spectra of NONP and CONP exhibited large semicircular arcs with high impedance values, indicating poor charge-transfer efficiency. In contrast, CNNC-1 showed relatively smaller semicircles and lower impedance, indicating enhanced interfacial charge transfer and reduced charge-transfer resistance. The reduced charge-transfer resistance strongly supports the formation of an efficient charge separation pathway in the CNNC-1 composite, resulting in synergistic photocatalytic activity.

### 2.4. Photocatalytic Degradation Performance

**Figure 8:**
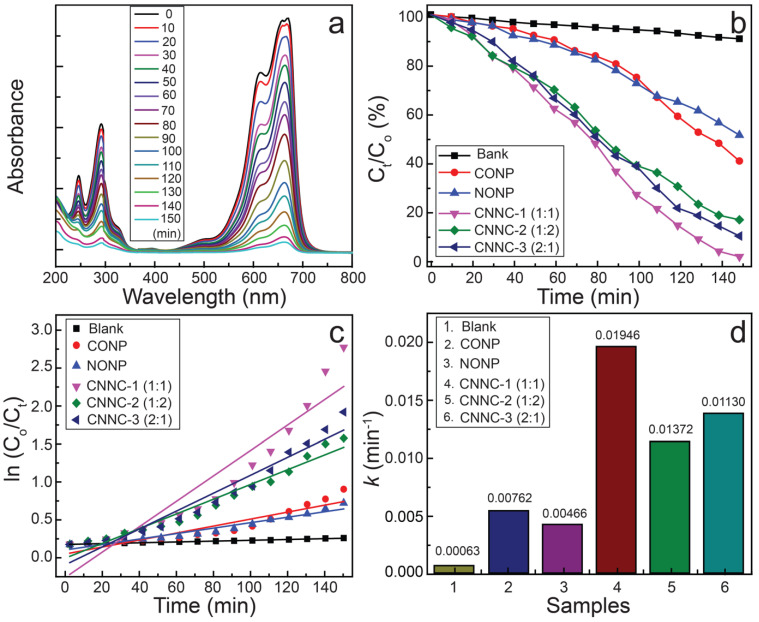
(**a**) UV–Vis absorbance spectra of residual MB after treatment with CNNC-1; (**b**) MB degradation plot; (**c**) pseudo-first-order kinetic plot; and (**d**) bar chart of rate constants for MB degradation using different photocatalysts under VL (initial MB concentration: 15 mg/L; catalyst dosage: 500 mg/L).

The photocatalytic efficiency of the prepared materials was investigated by monitoring the MB decomposition under VL exposure. The degradation process was assessed via UV–visible spectroscopy, and the results are presented in Figure 8a–d. Figure 8a shows the UV–Vis absorption profiles of MB over time using CNNC-1, illustrating a steady decrease in MB concentration under VL exposure.

Figure 8b compares the degradation efficiencies of various photocatalysts. After 150 min of irradiation, the observed MB degradation efficiencies were as follows: CNNC-1 (95.07%), CNNC-2 (80.30%), CNNC-3 (86.69%), CONP (54.56%), and NONP (46.20%). CNNC-1 exhibited the highest efficiency, which is attributed to the Co_3_O_4_/Nb_2_O_5_ heterojunction formation, extended VL absorption, and improved separation and extended lifetime of carriers, as confirmed by structural, morphological, optical, and PL analyses. Particularly, the XRD analysis confirmed the heterojunction formation between the orthorhombic phase NONP and the cubic phase CONP. The morphology revealed by SEM and TEM images showed high surface roughness and porosity in irregularly shaped NONP with uniform distribution of CONP, favoring increased surface area, directional charge transfer, and optimal absorption of light and pollutant molecules. Moreover, the Tauc plot indicated a low band gap of 2.39 eV for CNNC-1, supporting its extended visible light absorption range. The PL spectrum exhibited the lowest emission intensity for CNNC-1, suggesting an enhanced electron–hole separation rate, which further contributes to its superior photocatalytic activity. All of these collectively contributed to the enhanced ROS production and improved photocatalytic degradation efficiency of CNNC-1. In contrast, only 11.7% of MB was degraded under VL exposure without the presence of any catalyst (Figure S6). The UV–Vis spectra for MB degradation using CNNC-2, CNNC-3, CONP, and NONP are provided in Figure S7.

The photocatalytic degradation kinetics were analyzed using a pseudo-first-order kinetic scheme. Figure 8c shows that the decomposition profiles follow this model well. The rate constants (*k*) derived from the linear fitting of the plots are exhibited in Figure 8d: CNNC-1 (0.01946 min^−1^), CNNC-2 (0.01372 min^−1^), CNNC-3 (0.01130 min^−1^), CONP (0.00762 min^−1^), and NONP (0.00466 min^−1^). Among these, CNNC-1 displayed the highest rate constant, indicating the fastest photocatalytic degradation of MB. This synergistic result is supported by the highly increased surface area of CNNC-1 compared to the NONP and CONP, as shown in Table S2. The specific surface area is crucial for enhancing photocatalytic degradation by providing more active sites, improving light absorption, and promoting electron–hole separation [50]. In addition, the well-structured and porous surface morphology of the nanocomposites further increases the catalytic activity of the nanocomposite by enhanced light absorption, increased interaction with pollutant molecules, and promoted interfacial charge transfer, which is beneficial for enhanced production of ROS [66]. The lower rate constants for CNNC-2 and CNNC-3 may be attributed to excess amounts of Nb_2_O_5_ or Co_3_O_4_, which could hinder interfacial charge transfer or reduce active surface area. It has been reported that excessive loading of composite components results in surface overcoverage, which hinders light absorption at active sites and consequently reduces photocatalytic efficiency [42]. All kinetic fits yielded correlation coefficients (*R*^2^) greater than 0.89, as summarized in Table S4.

To further evaluate the photocatalytic versatility of CNNC-1, its performance was also tested against other pollutants—CR and TC—under identical VL illumination conditions. CNNC-1 achieved degradation efficiencies of 90.0% for CR and 72.6% for TC within 150 min, confirming its broad-spectrum photocatalytic capability. The corresponding kinetic plots and rate constants are provided in Figure S8. The rate constants observed for the degradation of MB, Congo red, and TC are in the decreasing order, 0.01946 > 0.01424 > 0.00807 min^−1^. The highest value of *k* observed for MB degradation is attributed to its simple molecular structure and photosensitizing nature. TC exhibited the lowest *k* value among the studied pollutants because of its structural complexity and non-photosensitizing effect. The azo bonds, moderately susceptible to oxidative attack, present in CR resulted in a moderate rate constant. Finally, a comparative analysis with previously reported photocatalysts (Table S5) shows that CNNC-1 exhibits superior degradation performance for various organic pollutants, highlighting its strong potential as a multipurpose photocatalyst for ecological remediation applications.

### 2.5. Bactericidal Activity

**Figure 9:**
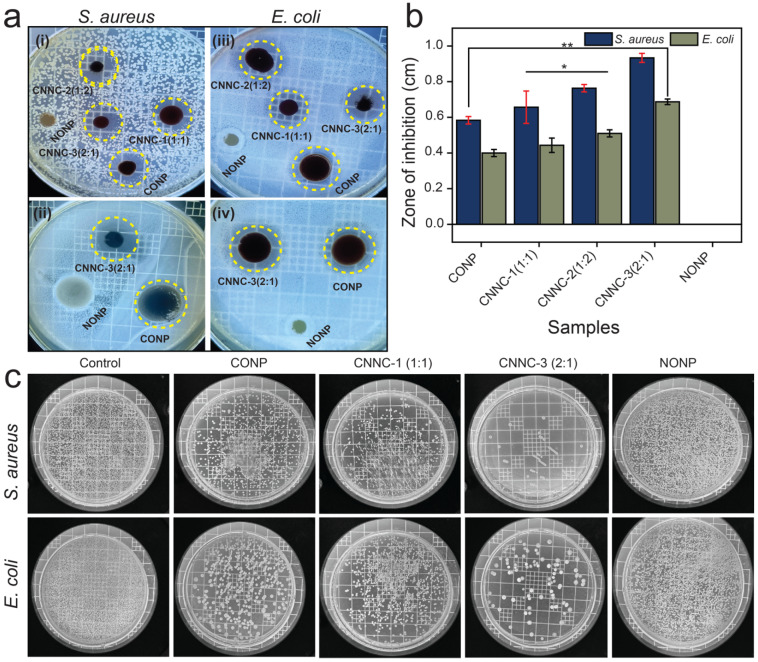
(**a**) Digital images of inhibition zones against *S aureus* for (i) all samples (ii) CONP, NONP, and CNNC-3, and against *E. coli* for (iii) all samples and (iv) CONP, NONP, and CNNC-3, (**b**) bar chart representing antibacterial activity, and (**c**) bacterial colonies visualized via plate count agar tests for *Staphylococcus aureus* and *Escherichia coli* under light irradiation. All deviations were taken as statistically significant designated as * *p* < 0.05 and ** *p* < 0.01.

The antibacterial efficiency of the individual components (CONP and NONP) and the synthesized CNNC nanocomposites was investigated against *Staphylococcus aureus* (Gram-positive) and *Escherichia coli* (Gram-negative), employing the standard agar-disc diffusion method. As shown in Figure 9a,b and summarized in Table 1, the CNNC nanocomposites exhibited significantly larger zones of inhibition (ZOI) compared to the individual nanoparticles, indicating enhanced bactericidal efficacy. All experiments were carried out in triplicate, and the ZOI values displayed in Table 1 represent the average of these measurements. The superior antibacterial efficiency of the CNNC composites is attributed to the combined effect of Co_3_O_4_ and Nb_2_O_5_, which likely enhances reactive oxygen species (ROS) production and disrupts bacterial cell membranes more effectively than the single-component systems. In addition, the increased surface area of the nanocomposite (Table S2) increases ROS production by increasing bacterial adhesion, improving light absorption, and promoting interfacial charge transfer. It also facilitates stronger interaction with bacterial membranes and enhances dispersion in aqueous environments. These combined effects contribute to more efficient bacterial inactivation through oxidative stress and membrane damage.

The ZOI results shown in Figure 9a,b demonstrate that Co_3_O_4_ exhibits notable antibacterial activity, primarily due to its redox-active nature. This activity promotes the production of ROS and the release of toxic Co^2+^ ions, both of which contribute to bacterial cell damage and death [49]. In contrast, Nb_2_O_5_ alone exhibits minimal antibacterial activity due to its chemical inertness and lack of inherent redox potential. However, when incorporated into a composite, its photocatalytic response can be enhanced, enabling ROS production through synergistic interactions [33]. Therefore, in this study, we used CONP as a positive control and NONP as a negative control.

The CNNC composites exhibited significantly enhanced antibacterial performance through two key mechanisms: (i) the production of ROS and (ii) the release of metal ions. Among the samples, CNNC-3 demonstrated the strongest antibacterial activity. This higher efficiency is attributed to the combined effect of the Co_3_O_4_/Nb_2_O_5_ heterojunction, which facilitates more efficient electron–hole separation and enhances ROS generation under light irradiation. Additionally, CNNC-3 contains a higher proportion of Co_3_O_4_, leading to greater Co^2+^ ion release and offering more active centers for interaction with bacterial cells. The enhanced antibacterial efficiency observed for CNNC-2 compared to CNNC-1 is attributed to the better dispersion of the CONP on the increased surface area of NONP, optimized interfacial interactions, and synergistic charge transfer at the heterojunction interface.

In the direct contact test (Figure 9c), a substantial reduction in viable bacterial colonies was observed for both *E. coli* and *S. aureus*. Notably, *S. aureus* exhibited greater susceptibility, which is likely due to the relatively thinner and more permeable peptidoglycan layer characteristic of Gram-positive bacteria, making them more vulnerable to oxidative and ionic damage compared to Gram-negative bacteria like *E. coli* [67].

**Figure 10:**
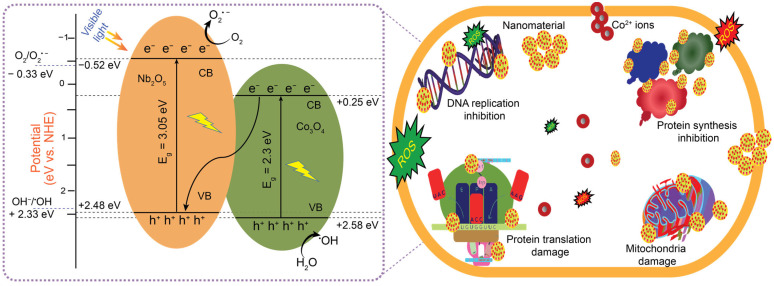
A schematic diagram illustrating the mechanism for antibacterial activity.

The promoted antibacterial efficiency of the CNNC composites can be ascribed to two primary mechanisms: (i) ROS generation under VL, producing species such as •OH, O_2_•^−^, and H_2_O_2_, which penetrate bacterial membranes, disrupt metabolic processes, and damage vital biomolecules, including lipids, proteins, and DNA, ultimately resulting in cell destruction [22,68], and (ii) Release of Co^2+^ ions, which can interact with negatively charged bacterial membranes, particularly with sulfhydryl (–SH) groups in membrane proteins, compromising membrane integrity, inhibiting cellular function, and promoting bacterial death [69]. In addition, a well-structured and porous morphology of nanocomposites increases surface area and enhances contact between the nanocomposites and bacterial cells, resulting in more efficient ROS-mediated bacterial membrane disruption. Moreover, specific morphologies, such as nanosheets, nanorods, or flower-like structures, can facilitate better light absorption and charge separation [49,66]. Furthermore, an optimized surface morphology supports uniform distribution of the heterojunction interfaces, ensuring consistent antibacterial effects. The diagram illustrating the mechanism of antibacterial activity is shown in Figure 10. NONP has no antibacterial activity on its own due to its large band gap, resulting in rapid recombination of charge carriers. However, it plays a synergistic role in CNNC nanocomposites by acting as an electron acceptor, which enhances the interfacial charge transfer, resulting in an increased lifespan of electron–hole pairs. This phenomenon delocalizes the concentration of electrons on NONP and holes on CONP, ultimately promoting the generation of ROS, which is beneficial for enhanced antibacterial activity. Furthermore, NONP contributes to the structural stability and biocompatibility of the composite, resulting in a more efficient and stable antibacterial material [38].

A comparison table for the antibacterial results obtained from our work with other relevant reported work is provided in Table S6. The comparison shows that our nanocomposite yields comparable results to those reported in the literature. Overall, these findings highlight the effectiveness of the CNNC nanocomposites—particularly CNNC-3—in controlling bacterial contamination through a dual-action mechanism, making them ideal candidates for applications in water disinfection and environmental health preservation.

### 2.6. Effect of Catalyst Dose, Pollutant Dose, Reusability, and Stability of Photocatalyst

The influence of catalyst amount on the MB decomposition using CNNC-1 was evaluated by altering the catalyst concentration from 30.0 to 70.0 mg/L. As shown in Figure S9a, MB removal efficiency increased from 82.2% to 95.1% as the dosage increased from 30.0 to 50.0 mg/L. This improvement is ascribed to the greater number of active centers and improved MB adsorption on the photocatalyst. However, further increasing the dosage to 70.0 mg/L showed a decrease in decomposition rate to 92.9%. This reduction is likely due to the light scattering and shielding effects caused by excess catalyst particles, which limit light penetration and reduce photoexcitation efficiency. Based on these results, 50.0 mg/L was identified as the optimal dosage for degrading 15.0 mg/L of MB.

The influence of initial MB concentration was also examined using CNNC-1 at concentrations of 10.0, 15.0, and 20.0 mg/L. As shown in Figure S9b, the removal efficiencies after 150 min of VL irradiation were 76.2%, 95.1%, and 81.5%, respectively. The highest degradation efficiency was achieved at 15.0 mg/L. At lower concentrations (10.0 mg/L), the limited number of MB molecules reduces the probability of effective interactions with the photocatalyst’s active sites. Conversely, at higher concentrations (20.0 mg/L), the photocatalyst surface may become saturated or blocked by excess MB molecules, hindering ROS generation and reducing degradation efficiency.

**Figure 11:**
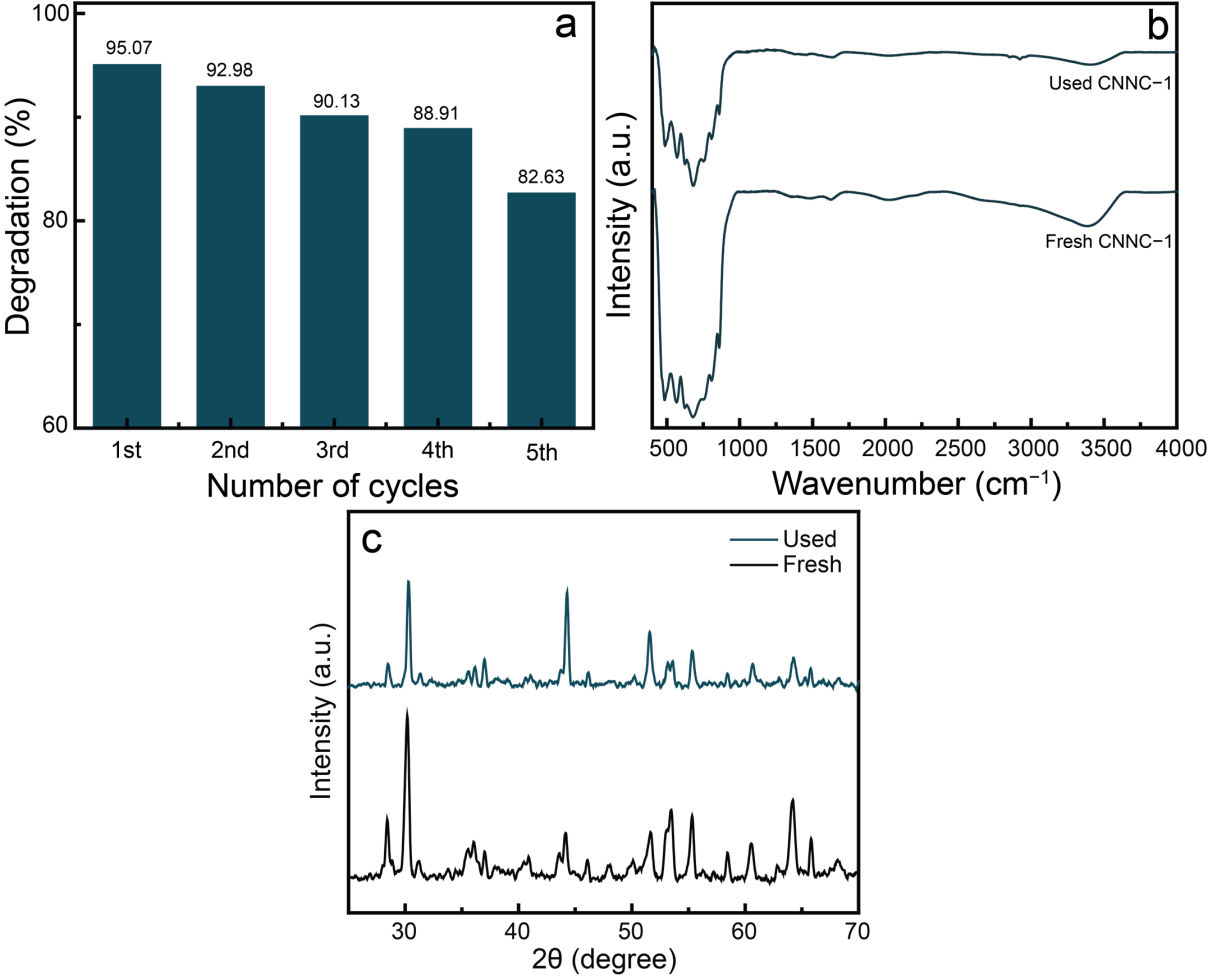
(**a**) Recyclability results of CNNC-1 for MB degradation, (**b**) FT-IR profiles, and (**c**) XRD spectra of fresh and used CNNC-1.

Reusability is a key factor in assessing the functional applicability of photocatalysts. Figure 11a presents the recycling performance of CNNC-1 over five successive degradation cycles of MB under identical conditions. The photocatalytic efficiency remained above 82% even after the fifth cycle, indicating excellent stability and recyclability. A minimal decline in performance after the fourth run may be due to photocatalyst loss during recovery or gradual surface fouling.

To additionally confirm the structural integrity of the photocatalyst, FTIR spectra and XRD were recorded after five cycles of reuse (Figure 11b,c). The characteristic absorption bands of CNNC-1 for FTIR remained unchanged, although a slight decrease in peak intensity was observed, likely due to surface adsorption of residual organic species or minor catalyst degradation. The XRD spectra (Figure 11c) also showed no significant change in the peak positions of the used samples compared to fresh CNNC-1, confirming the structural stability. These results confirm that the CNNC-1 nanocomposite retains its structural integrity and functional performance over multiple photocatalytic cycles.

### 2.7. Effects of pH_pzc_ and pH on Photocatalytic Decomposition

**Figure 12:**
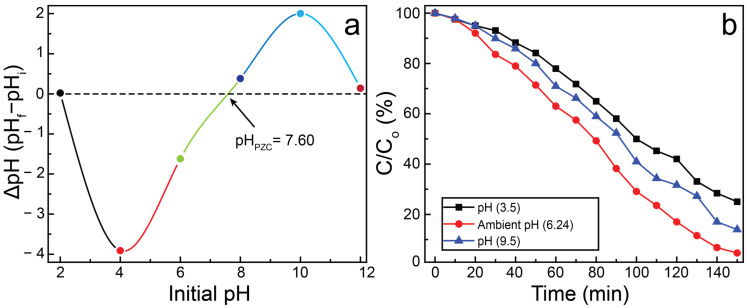
(**a**) Determination of pH_PZC_ for CNNC-1 and (**b**) influence of pH on the MB decomposition by CNNC-1.

The surface charge of a photocatalyst plays a crucial role in its interaction with charged pollutants and is governed by its point of zero charge (pH_pz__c_). The pH_pzc_ represents the pH at which the net surface charge of the photocatalyst becomes zero. For CNNC-1, the pH_pzc_ was estimated to be 7.60 utilizing the drift method (Figure 12a). At pH below the pH_pzc_, the surface of CNNC-1 becomes positively charged, whereas at pH above the pH_pzc_, the surface acquires a negative charge [70]. The photocatalytic performance of CNNC-1 for MB degradation was evaluated at different pH levels (3.5, 6.24, and 9.5), as demonstrated in Figure 12b. The highest degradation efficiency was exhibited at pH 6.24, while significantly reduced activity was noted at both acidic and alkaline extremes.

In alkaline conditions (pH = 9.5), the negatively charged catalyst surface may lead to excessive adsorption of MB, potentially blocking active sites. Additionally, the abundance of hydroxide ions (OH^−^) can hinder ROS generation by scavenging photogenerated holes, thereby reducing charge separation efficiency and photocatalytic activity. Under acidic conditions (pH = 3.5), the surface of CNNC-1 becomes positively charged. Since MB is a cationic dye, electrostatic repulsion arises between the similarly charged photocatalyst and dye molecules, suppressing adsorption and, thus, photocatalytic degradation. In contrast, at near-neutral pH (pH = 6.24), the surface charge of the sample is closer to neutral, minimizing repulsive interactions and enabling optimal adsorption of MB molecules. This promotes enhanced charge transfer, efficient ROS generation, and overall improved photocatalytic performance.

### 2.8. Trapping Experiments

To understand the predominant species involved in the MB decomposition, scavenger examinations were conducted using specific quenching agents: isopropyl alcohol (IPA) for •OH, potassium iodide (KI) for photogenerated h^+^, and benzoquinone (BQ) for O_2_•^−^. The findings are presented in Figure S10.

Without scavengers, CNNC-1 showed a decomposition efficiency of 95.1%. The addition of KI reduced the efficiency to 84.0%, indicating that h^+^ plays a minor but measurable role in the photodecomposition reaction. When BQ was added, the efficiency further declined to 67.9%, highlighting the important contribution of O_2_•^−^ radicals to the photocatalytic activity. Most notably, the introduction of IPA caused a sharp decline in MB degradation, with efficiency dropping to 29.2%. This pronounced reduction confirms that •OH radicals are the primary species involved in the degradation.

The order of inhibition observed—IPA > BQ > KI—suggests a reactivity hierarchy of •OH > O_2_•^−^ > h^+^. Although hydroxyl radicals are the primary contributors, both superoxide radicals and photogenerated holes also participate in the photodegradation mechanism. Overall, these findings confirm that all three species (•OH, O_2_•^−^, and h^+^) are generated during the photocatalytic reaction and synergistically contribute to the enhanced decomposition rate of MB.

### 2.9. Liquid Chromatography–Mass Spectrometry Analysis

To identify the decomposition products of MB and understand the decomposition mechanism, liquid chromatography–mass spectrometry (LC–MS) analysis was used. The corresponding spectra are provided in Figure S11. Since CNNC-1 demonstrated the most effective photocatalytic activity, the degraded MB solution treated with CNNC-1 was selected for analysis. An untreated MB solution was used as a control.

The LC–MS spectra of the degraded solution revealed the formation of several intermediate and final byproducts with characteristic mass-to-charge values, which aligned with those documented in earlier studies. The major *m*/*z* peaks were used to deduce the structural formulas of the photodegradation intermediates and end products, as demonstrated in Figure S12. The findings indicate that CNNC-1 effectively breaks down MB into smaller, non-toxic molecules. The final degradation products include water, carbon dioxide, nitrates, sulfates, acetic acid, and oxalic acid, demonstrating the mineralization of MB and confirming the environmental safety of the photocatalytic process.

### 2.10. Proposed Photocatalytic Mechanism

Upon exposure to VL, a photocatalyst absorbs photons with energy equal to or higher than its bandgap, promoting electrons from the VB to the CB and leaving behind holes in the VB. These photoexcited electron–hole pairs—also referred to as charge carriers—play a key role in redox reactions occurring at the photocatalyst surface.

*hν* + photocatalyst (CNNC-1) ⇌ (*e^−^CB* + *h^+^VB*) photocatalyst (3)

However, the photocatalytic efficiency of the semiconductor is largely based on the band edge potentials. The band edge potentials, *E*_CB_ and *E*_VB_, are influenced by many factors, including electronegativity, band gap, and free energy of the electron, and can be evaluated by using the following equations:

*E*_CB_ = χ − *E*_e_ − 0.5*E*_g_ (4)

*E*_VB_ = *E*_CB_ + *E*_g_ (5)

Here, χ is the electronegativity of the semiconductor (χ = 5.9 eV for Co_3_O_4_, χ _=_ 5.5 eV for Nb_2_O_5_ [71]); *E*_e_ is the free electron energy (*E*_e_ = 4.5 eV); *E*_VB_ and *E*_CB_ are the potentials of the VB and CB, respectively, and E_g_ is bandgap of Co_3_O_4_(2.3 eV), or Nb_2_O_5_(3.05 eV), as determined using Tauc equation (Figure 6). The determined values of the E_CB_ and E_VB_ for CONP and NONP using the above equations are +0.28 eV, −0.52 eV, and +2.58 eV, +2.48 eV vs. Normal Hydrogen Electrode, respectively.

Although Nb_2_O_5_ has a higher redox potential, its individual photocatalytic performance (NONP) is constrained by poor visible light absorption and a wide bandgap, which promotes rapid recombination of photogenerated carriers. In contrast, Co_3_O_4_ exhibits better performance than Nb_2_O_5_ due to its narrower bandgap and visible light sensitivity. However, the CNNC composites—especially CNNC-1—demonstrate substantially improved photocatalytic activity. This enhancement is ascribed to the heterojunction formation between CONP and NONP, which facilitates efficient interfacial charge transfer and subdues electron–hole recombination.

To explain this synergistic performance, a Z-scheme heterojunction mechanism is proposed rather than a conventional type-II heterojunction. In a Z-scheme system, the photoexcited electron in the CB of Co_3_O_4_ recombines with the hole in the VB of Nb_2_O_5_, allowing the high-energy electrons in Nb_2_O_5_’s CB and holes in Co_3_O_4_’s VB to remain available for redox reactions. This mechanism preserves strong redox potentials while enhancing charge separation, ultimately improving photocatalytic efficiency.

**Figure 13:**
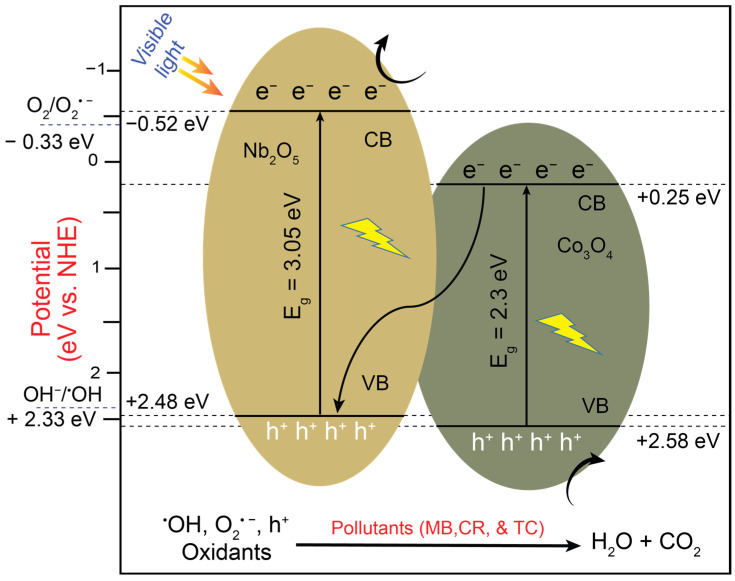
A schematic diagram illustrating the proposed Z-scheme photocatalytic mechanism in CNNC-1.

Figure 13 presents a schematic of the photocatalytic process proposed for CNNC-1 based on a Z-scheme heterojunction model. Upon VL exposure, electrons in both CONP and NONP are transferred from the VB to the CB, leaving behind corresponding holes in their VBs. The electrons in the CB of NONP are utilized to convert O_2_ into O_2_^•−^, since its reduction potential (−0.52 eV) is more negative than the reduction potential of O_2_/O_2_^•−^ (−0.33 eV vs. NHE). The holes in VB of CONP oxidize H_2_O or OH− into ^•^OH radicals due to its more positive potential (+2.58 eV) in comparison to the oxidation potential of H_2_O/OH^•^ (+2.33 eV vs. NHE). However, the CB electrons of CONP can neither reduce O_2_ nor migrate to the CB of NONP due to its low CB potential (+0.25 eV). Therefore, these electrons are migrated from the CB of CONP to the VB of NONP through the Z-scheme at the heterojunction interface. This phenomenon enhances the separation of charge carriers, delocalizes the charged species at the redox sites, and eventually improves the photocatalytic efficiency of the catalyst. The extended lifetime of the electron–hole pairs through improved charge separation and improved efficiency of the photocatalyst aligns with the results from the PL, EIS, and UV–Vis curves shown in Figure 7a,b and Figure 8. The ^•^OH and O_2_^•−^ radicals play an active role in the MB decomposition, as demonstrated by the scavenger tests in Section 2.8. The detailed steps of the reaction involved in the decomposition of pollutants such as MB, CR, and TC are provided as follows from Equations (6)–(14):

CNNC-1 + hν → Nb_2_O_5_ (e^−^_CB_ + h^+^ _VB_) + Co_3_O_4_ (e^−^_CB_ + h^+^ _VB_) (6)

Upon illumination with light, photoexcited electrons are produced, with h^+^ in the VB (6). The photoinduced electrons and holes interact with oxygen and water to generate highly reactive hydroxyl and superoxide radicals (Equations (7) and (8)). Oxygen adsorbed on the surface captures electrons, causing the formation of superoxide radicals (O_2_^•−^). These superoxide radicals then interact with protons (H^+^) and oxygen (O_2_) to produce hydrogen peroxide (Equations (9) and (10)). The hydrogen peroxide subsequently reacts with electrons, O_2_^•−^, yielding •OH and OH^−^ (Equations (11) and (12)). Meanwhile, the hydrogen peroxide absorbs light, generating additional ^•^OH (Equation (13)). Both ^•^OH and O_2_^•−^ then react with pollutants, resulting in the final decomposition products, such as water and carbon dioxide (Equation (14)).

Nb_2_O_5_ (e^-^_CB_ + O_2_) → ^•^O_2_^−^ (7)

Co_3_O_4_ (h^+^_VB_ + H_2_O) → ^•^OH + H^+^ (8)

^•^O_2_^−^ + H^+^ → HO_2_^•^ (9)

HO_2_^•^ + HO_2_^•^ → H_2_O_2_ + O_2_ (10)

H_2_O_2_ + e^−^ → ^•^OH + OH^−^ (11)

^•^O_2_^−^ + H_2_O_2_ → O_2_ + HO^•^ + OH^−^ (12)

H_2_O_2_ + hν → 2^•^OH (13)

(^•^OH, ^•^O_2_^−^) + Organic pollutants → Degradation products (H_2_O, CO_2_) (14)

Coupling Co_3_O_4_ with a wide-bandgap semiconductor like Nb_2_O_5_ offers a promising strategy to enhance visible-light photocatalysis. This combination improves redox site availability, reduces charge carrier recombination, and facilitates efficient charge migration across the heterojunction interface. However, one challenge in fabricating such heterostructures lies in maintaining the structural integrity of both Co_3_O_4_ and Nb_2_O_5_, as these oxides may undergo phase transformations or surface changes during synthesis and operation that could alter their catalytic performance [29,43].

## 3. Experimental

### 3.1. Chemicals

All chemicals were employed as procured. The following reagents were utilized in the synthesis and photocatalytic experiments: ammonium niobate(V) oxalate hydrate (99.99%, Sigma-Aldrich, St. Louis, MO, USA), cobalt(II) acetate tetrahydrate (ACS reagent, ≥98.0%, Sigma-Aldrich), ethyl alcohol (200 proof, ACS reagent, ≥99.5%, Sigma-Aldrich), sodium chloride (NaCl, ACS reagent, ≥99.0%, Sigma-Aldrich), sodium hydroxide (NaOH, reagent grade, ≥98.0%, Sigma-Aldrich), hydrochloric acid (HCl, Sigma-Aldrich), isopropyl alcohol (IPA, ≥99.7%, Sigma-Aldrich), benzoquinone (BQ, ≥98%, reagent grade), potassium iodide (KI, ≥99%, Sigma-Aldrich), MB (high purity, Alfa Aesar, Haverhill, MA, USA), CR (high purity, Sigma-Aldrich), and TC (98–102%, HPLC grade, Sigma-Aldrich).

### 3.2. Synthesis of CNNC

**Scheme 1:**
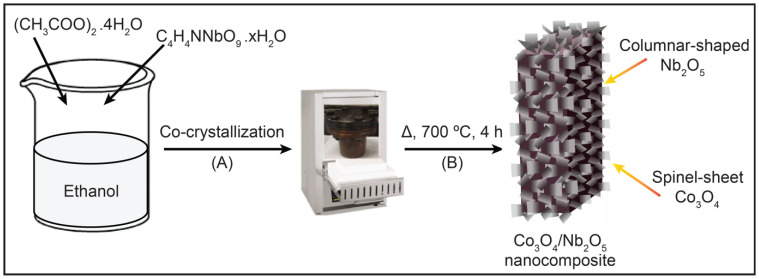
Scheme for the synthesis of CNNC nanocomposites via (A) co-crystallization and (B) annealing.

CNNC was prepared via a simple co-crystallization technique and subsequent high-pressure annealing. Initially, stoichiometric amounts (1.0 g each) of cobalt(II) acetate tetrahydrate ((CH_3_COO)_2_Co·4H_2_O) and ammonium niobate(V) oxalate hydrate (C_4_H_4_NNbO_9_·xH_2_O) were well mixed and ground in a mortar for 10 min to achieve a uniform blend. The resulting mixture was sonicated in 50 mL of anhydrous ethyl alcohol and stirred continuously for 4 h to promote uniform dispersion of the metal precursors. The suspension was then located in an oven and kept at 70 °C for 24 h to facilitate solvent evaporation and precursor co-crystallization. After drying, the solid residue was reground and transferred into a quartz crucible with a lid. The crucible was placed in a stainless-steel SUS314 container, sealed with a copper gasket to ensure a controlled high-pressure environment (~2–3 atm) and prevent oxygen ingress during calcination. The sealed system was then annealed at 700 °C for 4 h with a heating rate of 7 °C/min using a muffle furnace (KSL-1100X-S-LD, MTI Corporation, Richmond, CA, USA). Post-calcination, the product was rinsed multiple times with water and ethanol to remove residual byproducts, followed by drying overnight at 70 °C. The final composite, labeled CNNC-1, was stocked in an airtight container for characterization and testing. To investigate the effect of composition, two additional nanocomposites—CNNC-2 and CNNC-3—were prepared using the same procedure but with precursor weight ratios of 1:2 and 2:1 (Co_3_O_4_:Nb_2_O_5_), respectively. The overall synthesis procedure is shown in Scheme 1.

### 3.3. Synthesis of CONP and NONP

The spinel Co_3_O_4_ nanoparticles (CONP) and columnar-shaped Nb_2_O_5_ nanoparticles (NONP) were synthesized individually using the same procedure discussed in Section 3.2, employing cobalt(II) acetate tetrahydrate and ammonium niobate(V) oxalate hydrate as precursors. These materials were prepared as control samples to compare with the Co_3_O_4_/Nb_2_O_5_ nanocomposites in evaluating photocatalytic performance.

### 3.4. Characterization Techniques

The structural, morphological, optical, and compositional characteristics of the prepared materials were systematically examined using a suite of analytical techniques. Detailed descriptions of the instrumentation and methods are provided in the Supplementary Information.

### 3.5. Antibacterial Activity

The antibacterial efficiency of both the individual (CONP, NONP) and composite (CNNC) samples was examined by measuring the inhibition zones against two representative bacterial strains: *Staphylococcus aureus* (Gram-positive, ATCC 29231) and *Escherichia coli* (Gram-negative, ATCC 52922). Bacterial cultures were grown in Tryptic Soy Broth (TSB) at 37 °C for 12 h under shaking conditions (100 rpm). Prior to incubation, 1 × 10^7^ colony-forming units of bacterial dispersion were evenly spread onto Tryptic Soy Agar (TSA) plates.

Each nanocomposite was first exposed to UV light for 12 h to ensure sterilization. A colloidal suspension was then prepared by dispersing the nanomaterials at a concentration of 5 mg/mL in 500 µL of deionized water. This suspension was applied dropwise to the inoculated agar plates and incubated at 37 °C for an additional 12 h. The resulting inhibition zones were photographed using a digital camera and measured to determine bactericidal efficiency. Additionally, dynamic contact tests were performed to quantitatively assess antibacterial efficacy. In this method, 1 mL of bacterial suspension (CFU = 1.5 × 10^4^/mL) was incubated with 5 µL of nanocomposite material in sterile vials. These mixtures were shaken at 100 rpm for 60 min at 37 °C. Following incubation, serial dilutions (dilution factor = 10^4^, performed in triplicate) were prepared using sterile double-distilled water. The diluted suspensions were plated on TSA and incubated at 37 °C for 24 h. The resulting bacterial colonies were imaged with a digital camera for quantitative analysis.

### 3.6. Photocatalytic Experiments

Photocatalytic investigations were carried out to examine the degradation efficiency of the synthesized composites—particularly CNNC-1—toward MB, CR, and TC on VL exposure. A 300 W Xenon lamp (OSRAM XBO R 300W/60C, Osram, Munich, Germany) equipped with a UV cut-off filter (λ < 400 nm) was employed as the source to ensure that only visible light was utilized.

In each experiment, 50.0 mg of CNNC-1 (with a Co_3_O_4_:Nb_2_O_5_ ratio of 1:1) was introduced to 100 mL of a solution containing 15.0 mg/L of the target pollutant (MB, CR, or TC). The mixture was ultrasonicated for 30 min to ensure uniform dispersion and then stirred magnetically for an additional 30 min. The dispersion was kept in the dark for 1 h to establish adsorption–desorption equilibrium between the photocatalyst and the pollutant. Photocatalytic degradation was performed under ambient conditions without the addition of external oxidizing agents. Throughout the reaction, 3 mL aliquots were collected at 10-minute intervals and investigated via UV–Vis spectrophotometer to study changes in absorbance. All experiments were performed under similar conditions to ensure consistency. The decomposition efficiency (*η*) was determined using Equation (15):

(15) $$\eta \left(\%\right)=\frac{C_{0}-C_{t}}{C_{0}}\times 100$$

where *η* denotes the degradation efficiency, and *C*_0_ and *C_t_* are concentrations of the pollutant at illumination time 0 and *t*, respectively. The Langmuir–Hinshelwood pseudo-first-order kinetic equation (Equation (16)) was applied to assess the rate constant of the photocatalytic reactions for pollutant degradation using various photocatalysts, as previously described [72].

(16) $$k=\ln\frac{C_{0}∕C_{t}}{t}$$

### 3.7. Reusability Assessment

The recyclability and robustness of the best-performing photocatalyst, CNNC-1, were evaluated over five successive photocatalytic cycles using MB as the model pollutant. After each run, the photocatalyst was recovered by centrifugation, thoroughly cleaned with deionized water to eliminate residual contaminants, and dried at 70 °C overnight. The dried material was then reused in the subsequent cycle under identical experimental conditions to evaluate its sustained photocatalytic performance.

## 4. Conclusions

CNNC heterojunction nanocomposites were prepared through a one-step co-crystallization method followed by high-pressure annealing. The structural, morphological, and chemical characteristics of the nanocomposites were thoroughly characterized using several analytical tools. The photocatalytic and antibacterial properties of the prepared samples were systematically assessed. Among the synthesized samples, CNNC-1 exhibited outstanding photocatalytic performance, achieving 95.1% decomposition of MB within 150 min under VL illumination. The corresponding reaction rate constant was 367% of that of CONP and 466% of that of NONP, underscoring the enhanced activity due to heterojunction formation. Additionally, CNNC-1 demonstrated broad-spectrum photocatalytic efficiency, degrading 90.0% of Congo red and 72.6% of tetracycline under identical conditions. Reusability tests confirmed that CNNC-1 maintained over 82% of its initial activity after five cycles, with minimal performance loss. Structural stability was further supported by XRD analysis and FT-IR analysis of the used photocatalyst. LC–MS analysis confirmed the degradation of organic pollutants into benign end products such as H_2_O, CO_2_, nitrates, sulfates, and low-molecular-weight acids. In terms of antibacterial activity, CNNC-3 showed the highest efficacy, producing inhibition zones of 9.3 mm against *Staphylococcus aureus* and 6.8 mm against *Escherichia coli*. This promoted antibacterial efficiency can be ascribed to the higher content of Co_3_O_4_, which facilitates a greater release of toxic Co^2+^ ions and reactive oxygen species. The remarkable photocatalytic and antimicrobial activities of the CNNC nanocomposites are the result of an efficient Z-scheme heterojunction formation, which enhances extended light absorption, improved charge separation, enhanced ROS generation, and retention of strong redox potentials. These findings collectively demonstrate the bifunctional potential of CNNC composites for environmental remediation. Compared to the previously reported Co_3_O_4_ and Nb_2_O_5_-based nanocomposites, our work stands out due to its facile synthesis method, bifunctional applicability, and efficient charge separation via the Z-scheme mechanism.

These results provide valuable insight into the design of VL-driven multifunctional photocatalysts. Future investigations will focus on evaluating the composites in real wastewater systems, toxicity analysis of treated effluents, and assessment of long-term operational stability for degradation of emerging pollutants and assessing their antibacterial performance against a broader range of microbial strains.

## Figures and Tables

**Table 1:** Zone of inhibition of the prepared samples.

Samples	*S. aureus* (mm)	*E. coli* (mm)
CONP	5.8	4.0
CNNC-1 (1:1)	6.2	4.8
CNNC-2 (1:2)	7.8	4.9
CNNC-3 (2:1)	9.3	6.8
NONP	0	0

## Data Availability

The original contributions presented in this study are included in the article/Supplementary Material. Further inquiries can be directed to the corresponding author.

## Supplementary Materials

The following supporting information can be downloaded at https://www.mdpi.com/article/10.3390/molecules30122561/s1. Figure S1: Particle size distribution plot for (a) CNNC-1, (b) CNNC-2, and (c) CNNC-3. Figure S2: (a) FE-SEM image, (b) EDS layered image, (c–e) elemental distribution (O, Nb, and Co), and (f) EDS spectrum of CNNC-1. Figure S3: XPS spectra: (a) Co-2*p* in individual CONP and (b) Nb-3*d* in individual NONP. Figure S4: XPS spectra: Co-2*p*, Nb-3*d*, and O-1s in (a–c) CNNC-2 and (d–f) CNNC-3. Figure S5. Tauc plot of CNNC-3. Figure S6: Degradation spectra of MB in the absence of a photocatalyst. Figure S7: UV-Vis spectra of MB degradation for (a) CNNC-2, (b) CNNC-3, (c) CONP, and (d) NONP. Figure S8: Degradation plot and kinetic plot of CNNC-1 for (a,b) CR and (c,d) TC. Figure S9: MB degradation bar diagram of CNNC-1 for the effect of (a) catalyst dose and (b) concentration. Figure S10: Effect of charge carrier scavengers for MB degradation using CNNC-1. Figure S11: LC-MS spectra of (a) fresh MB and (b) degraded MB using CNNC-1. Figure S12: Proposed degradation pathway of MB. Table S1: The average particle size (nm) of the Nanocomposite. Table S2: Comparison of the BET surface area and pore volume (BJH) of samples. Table S3: The structural parameters of synthesized materials. Table S4. R^2^ value of the pseudo-first-order reaction of the prepared materials. Table S5: Comparison of the photocatalytic efficiency of Co_3_O_4_/Nb_2_O_5_ composites and related composite materials. Table S6: Comparison of the antibacterial activity of Co_3_O_4_/Nb_2_O_5_ composites and relevant composite materials. Characterization techniques detail, SEM image and EDS mapping of CNNC-1, XPS spectra of Co-2p in CONP and Nb-3d in NONP, Tauc plot of CNNC-3, photolysis plot of MB, photodegradation curve for MB using CNNC-2, CNNC-3, CONP, and NONP, degradation plot and kinetic plot of CNNC-1 for CR and TC, catalyst dose and concentration-dependent bar diagram of CNNC-1 for MB, MB degradation plot for scavengers effect, LCMS spectra for fresh and degraded MB, proposed degradation pathway of MB, Table S1, for R2 value of kinetic plots, Table S2 for comparison of photocatalytic efficiency of CNNC-1 with related composite materials, References [73,74,75,76,77,78,79,80,81,82,83,84] are cited in Supplementary Materials.

## References

[1] Nicoloff H., Hjort K., Levin B.R., Andersson D.I. (2019). The high prevalence of antibiotic heteroresistance in pathogenic bacteria is mainly caused by gene amplification. Nat. Microbiol..

[2] Liu H., Ma S., Shao L., Liu H., Gao Q., Li B., Fu H., Fu S., Ye H., Zhao F. (2020). Defective engineering in graphitic carbon nitride nanosheet for efficient photocatalytic pathogenic bacteria disinfection. Appl. Catal. B.

[3] Chakravarty S., Massé E. (2019). RNA-dependent regulation of virulence in pathogenic bacteria. Front. Cell. Infect. Microbiol..

[4] Karim M.N., Singh M., Weerathunge P., Bian P., Zheng R., Dekiwadia C., Ahmed T., Walia S., Della Gaspera E., Singh S. (2018). Visible-light-triggered reactive-oxygen-species-mediated antibacterial activity of peroxidase-mimic CuO nanorods. ACS Appl. Nano Mater..

[5] Makvandi P., Wang C.y., Zare E.N., Borzacchiello A., Niu L.n., Tay F.R. (2020). Metal-based nanomaterials in biomedical applications: Antimicrobial activity and cytotoxicity Aspects. Adv. Funct. Mater..

[6] Gogate P.R., Pandit A.B. (2004). A review of imperative technologies for wastewater treatment I: Oxidation technologies at ambient conditions. Adv. Environ. Res..

[7] Low J., Yu J., Ho W. (2015). Graphene-based photocatalysts for CO_2_ reduction to solar fuel. J. Phys. Chem. Lett..

[8] Shrestha S., Sapkota K.P., Lee I., Islam M.A., Pandey A., Gyawali N., Akter J., Mohan H., Shin T., Jeong S. (2022). Carbon-based ternary nanocomposite: Bullet type ZnO-SWCNT-CuO for substantial solar-driven photocatalytic decomposition of aqueous organic contaminants. Molecules.

[9] Khodabandeloo F., Shahsavarifar S., Nayebi B., Niavol K.P., Nayebi B., Varma R.S., Cha J.H., Jang H.W., Kim D., Shokouhimehr M. (2023). Applications of nanostructured semiconductor photocatalysts for the decontamination of assorted pollutants from wastewater. Inorg. Chem. Commun..

[10] Jabbar Z.H., Esmail Ebrahim S. (2022). Recent advances in nano-semiconductors photocatalysis for degrading organic contaminants and microbial disinfection in wastewater: A comprehensive review. Environ. Nanotechnol. Monit. Manag..

[11] Li X., Zhu J., Li H. (2012). Comparative study on the mechanism in photocatalytic degradation of different-type organic dyes on SnS_2_ and CdS. Appl. Catal. B.

[12] Mandal S., Adhikari S., Pu S., Wang X., Kim D.H., Patel R.K. (2019). Interactive Fe_2_O_3_/porous SiO_2_ nanospheres for photocatalytic degradation of organic pollutants: Kinetic and mechanistic approach. Chemosphere.

[13] Gyawali N., Kandel R., Lee I., Shrestha S., Pandey A., Akter J., Hahn J.R. (2024). Silver decoration of Cr_2_O_3_ nanoparticles: Facile preparation of Cr_2_O_3_ nanoparticles and Ag–Cr_2_O_3_ nanocomposites and characterization of their antibacterial activity and ability to photocatalytically degrade organic wastes under visible light. J. Photochem. Photobiol. A.

[14] Zhang Q.-Q., Ying G.-G., Pan C.-G., Liu Y.-S., Zhao J.-L. (2015). Comprehensive evaluation of antibiotics emission and fate in the river basins of china: Source analysis, multimedia modeling, and linkage to bacterial resistance. Environ. Sci. Technol..

[15] Shrestha S., Amatya S.P. (2021). Biosynthesis of manganese nanoparticles (MnNPs) from brassica oleraceae (cabbage leaves) and its antibacterial activity. Asian J. Chem. Sci..

[16] Crini G., Lichtfouse E. (2019). Advantages and disadvantages of techniques used for wastewater treatment. Environ. Chem. Lett..

[17] Fujishima A., Honda K. (1972). Electrochemical photolysis of water at a semiconductor electrode. Nature.

[18] Raj Kumar R., Ramesh R. (2015). Synthesis, molecular structure and electrochemical properties of nickel(ii) benzhydrazone complexes: Influence of ligand substitution on DNA/protein interaction, antioxidant activity and cytotoxicity. RSC Adv..

[19] Gyawali N., Lee I., Shrestha S., Acharya S., Zahid A., Kim K., Sapkota K.P., Hahn J.R. (2025). MOF-derived in situ confinement of copper/copper oxide nanoparticles inside a carbon tube: A facile morphologically controlled synthesis strategy for superior visible-light-driven photocatalytic efficiency. Ind. Eng. Chem. Res..

[20] Low J., Yu J., Jaroniec M., Wageh S., Al-Ghamdi A.A. (2017). Heterojunction photocatalysts. Adv. Mater..

[21] Wang S., Yang X., Zhang X., Ding X., Yang Z., Dai K., Chen H. (2017). A plate-on-plate sandwiched Z-scheme heterojunction photocatalyst: BiOBr-Bi_2_MoO_6_ with enhanced photocatalytic performance. Appl. Surf. Sci..

[22] Joshi B., Regmi C., Dhakal D., Gyawali G., Lee S.W. (2018). Efficient inactivation of staphylococcus aureus by silver and copper loaded photocatalytic titanate nanotubes. Prog. Nat. Sci. Mater. Int..

[23] Hao Q., Mo Z., Chen Z., She X., Xu Y., Song Y., Ji H., Wu X., Yuan S., Xu H. (2018). 0D/2D Fe_2_O_3_ quantum dots/g-C_3_N_4_ for enhanced visible-light-driven photocatalysis. Colloids Surf. A.

[24] Luo J., Zhang S., Sun M., Yang L., Luo S., Crittenden J.C. (2019). A critical review on energy conversion and environmental remediation of photocatalysts with remodeling crystal lattice, surface, and interface. ACS Nano.

[25] Li T., Nam G., Liu K., Wang J.-H., Zhao B., Ding Y., Soule L., Avdeev M., Luo Z., Zhang W. (2022). A niobium oxide with a shear structure and planar defects for high-power lithium ion batteries. Energy Environ. Sci..

[26] Dsouki N.A., de Lima M.P., Corazzini R., Gascon T.M., Azzalis L.A., Junqueira V.B., Feder D., Fonseca F.L. (2014). Cytotoxic, hematologic and histologic effects of niobium pentoxide in Swiss mice. J. Mater. Sci Mater. Med..

[27] Hong Y., Li C., Zhang G., Meng Y., Yin B., Zhao Y., Shi W. (2016). Efficient and stable Nb_2_O_5_ modified g-C_3_N_4_ photocatalyst for removal of antibiotic pollutant. Chem. Eng. J..

[28] Jones B.M.F., Mamba G., Maruthamani D., Muthuraj V. (2022). Honeycomb Nb_2_O_5_/RGO wrapped on MoO_3_ nanorods for visible light-driven degradation of sulfasalazine and ciprofloxacin in water. Colloids Surf. A.

[29] Su K., Liu H., Gao Z., Fornasiero P., Wang F. (2021). Nb_2_O_5_-based photocatalysts. Adv. Sci..

[30] Aimaiti G., Ma Y., Shi Y., Wang X., Wang S., Wang Z., Li Y., Li J., Qi X., Chen X. (2023). Nb_2_O_5_/red phosphorus S-scheme heterojunction photocatalyst for removal of organic contaminant and Cr(VI): Electrochemical performance and mechanism. Mater. Sci. Semicond. Process..

[31] Nogueira A.E., Lopes O.F., Neto A.B.S., Ribeiro C. (2017). Enhanced Cr(VI) photoreduction in aqueous solution using Nb_2_O_5_/CuO heterostructures under UV and visible irradiation. Chem. Eng. J..

[32] Wu J., Li J., Liu J., Bai J., Yang L. (2017). A novel Nb_2_O_5_/Bi_2_WO_6_ heterojunction photocatalytic oxidative desulfurization catalyst with high visible light-induced photocatalytic activity. RSC Adv..

[33] Gao B., Fu J., Huo K., Zhang W., Xie Y., Chu P.K. (2011). Quasi-aligned Ag–Nb_2_O_5_ nanobelt arrays with enhanced photocatalytic and antibacterial activities. J. Am. Ceram. Soc..

[34] Ren X., Shi J., Duan R., Di J., Xue C., Luo X., Liu Q., Xia M., Lin B., Tang W. (2022). Construction of high-efficiency CoS@Nb_2_O_5_ heterojunctions accelerating charge transfer for boosting photocatalytic hydrogen evolution. Chin. Chem. Lett..

[35] Tian M., Su F., Wang Z., Xiao Y., Zhang Y., Gao Y., Zhao Q., Jin X., Li X., Xie H. (2025). Constructing a stable CdS/Nb_2_O_5_ Z-scheme heterojunction for efficient photocatalytic conversion of CO_2_. Mol. Catal..

[36] Wang Y., Kong X., Jiang M., Zhang F., Lei X. (2020). A Z-scheme ZnIn_2_S_4_/Nb_2_O_5_ nanocomposite: Constructed and used as an efficient bifunctional photocatalyst for H_2_ evolution and oxidation of 5-hydroxymethylfurfural. Inor. Chem. Front..

[37] Souza L.V.S., Pavanello L., Picolo M.Z.D., Kury M., Matos I., Cogo-Muller K., Esteban Florez F.L., Cavalli V. (2023). Mechanical and antibacterial properties of an experimental flowable composite containing Nb_2_O_5_ and NF_TiO_2_ nanoparticles. J. Mech. Behav. Biomed. Mater..

[38] Madhavi B., Siva Sesha Reddy A., Syam Prasad P., Prasad A., Pavani Koteswari Devi P., Ravi Kumar V., Veeraiah N. (2021). The impact of Nb_2_O_5_ on in-vitro bioactivity and antibacterial activity of CaF_2_–CaO–B_2_O_3_–P_2_O_5_–SrO glass system. Ceram. Int..

[39] Safavi M.S., Khalil-Allafi J., Restivo E., Ghalandarzadeh A., Hosseini M., Dacarro G., Malavasi L., Milella A., Listorti A., Visai L. (2023). Enhanced in vitro immersion behavior and antibacterial activity of NiTi orthopedic biomaterial by HAp-Nb_2_O_5_ composite deposits. Sci. Rep..

[40] Chauhan A., Kumar R., Devi S., Sonu, Raizada P., Singh P., Ponnusamy V.K., Sudhaik A., Mishra A.K., Selvasembian R. (2024). Recent advances on Co_3_O_4_-based nanostructure photocatalysis: Structure, synthesis, modification strategies, and applications. Surf. Interfaces.

[41] Kumar S., Kaur G., Rawat M., Tsang Y.F., Lin K.-Y., Kim K.-H. (2022). Potential of piper betle@Co_3_O_4_ nanoparticles as high-performance photocatalysts for the removal of industrial dyes. J. Clean. Prod..

[42] Wang Y., Zhu C., Zuo G., Guo Y., Xiao W., Dai Y., Kong J., Xu X., Zhou Y., Xie A. (2020). 0D/2D Co_3_O_4_/TiO_2_ Z-Scheme heterojunction for boosted photocatalytic degradation and mechanism investigation. Appl. Catal. B.

[43] Subagyo R., Yudhowijoyo A., Sholeha N.A., Hutagalung S.S., Prasetyoko D., Birowosuto M.D., Arramel A., Jiang J., Kusumawati Y. (2023). Recent advances of modification effect in Co_3_O_4_-based catalyst towards highly efficient photocatalysis. J. Colloid Interface Sci..

[44] Tran V.A., Phung T.K., Le V.T., Vo T.K., Nguyen T.T., Nguyen T.A.N., Viet D., Hieu V.Q., Vo T.-T.T. (2021). Solar-light-driven photocatalytic degradation of methyl orange dye over Co_3_O_4_-ZnO nanoparticles. Mater. Lett..

[45] Raja V., Puvaneswaran S.K., Swaminathan K. (2017). Unique and hierarchically structured novel Co_3_O_4_/NiO nanosponges with superior photocatalytic activity against organic contaminants. Front. Mater. Sci..

[46] Shen C.-H., Wen X.-J., Fei Z.-H., Liu Z.-T., Mu Q.-M. (2020). Visible-light-driven activation of peroxymonosulfate for accelerating ciprofloxacin degradation using CeO_2_/Co_3_O_4_ p-n heterojunction photocatalysts. Chem. Eng. J..

[47] Malefane M.E., Feleni U., Kuvarega A.T. (2020). Cobalt (II/III) oxide and tungsten (VI) oxide p-n heterojunction photocatalyst for photodegradation of diclofenac sodium under visible light. J. Environ. Chem. Eng..

[48] Yousefi S.R., Alshamsi H.A., Amiri O., Salavati-Niasari M. (2021). Synthesis, characterization and application of Co/Co_3_O_4_ nanocomposites as an effective photocatalyst for discoloration of organic dye contaminants in wastewater and antibacterial properties. J. Mol. Liq..

[49] Wang X., Shao Y., Yao C., Huang L., Song W., Yang X., Zhang Z. (2025). 2D/2D Co_3_O_4_/BiOCl nanocomposite with enhanced antibacterial activity under full spectrum: Synergism of mesoporous structure, photothermal effect and photocatalytic reactive oxygen species. J. Colloid Interface Sci..

[50] Liyanaarachchi H., Thambiliyagodage C., Jayanetti M., Ekanayake G., Wijayawardana S., Samarakoon U. (2025). The photocatalytic and antibacterial activity of graphene oxide coupled CoO_x_ /MnO_x_ nanocomposites. Environ. Technol. Innov..

[51] Guo M., Xu K., Qu Y., Zeng F., Yuan C. (2018). Porous Co_3_O_4_/CoS_2_ nanosheet-assembled hierarchical microspheres as superior electrocatalyst towards oxygen evolution reaction. Electrochim. Acta.

[52] Li Z., Yu X.-Y., Paik U. (2016). Facile preparation of porous Co_3_O_4_ nanosheets for high-performance lithium ion batteries and oxygen evolution reaction. J. Power Sources.

[53] Palanisamy G., Bhuvaneswari K., Srinivasan M., Vignesh S., Elavarasan N., Venkatesh G., Pazhanivel T., Ramasamy P. (2021). Two-dimensional g-C_3_N_4_ nanosheets supporting Co_3_O_4_-V_2_O_5_ nanocomposite for remarkable photodegradation of mixed organic dyes based on a dual Z-scheme photocatalytic system. Diam. Relat. Mater..

[54] Janani B., Syed A., Al-Shwaiman H.A., Alkhulaifi M.M., Elgorban A.M., Khan S.S. (2021). Performance analysis of novel Bi_6_Cr_2_O_15_ coupled Co_3_O_4_ nano-heterostructure constructed by ultrasonic assisted method: Visible-light driven photocatalyst and antibacterial agent. Colloids Surf. A.

[55] Khatoon R., Guo Y., Attique S., Khan K., Treen A.K., Haq M.U., Tang H., Chen H., Tian Y., Nisar M. (2020). Facile synthesis of α-Fe_2_O_3_/Nb_2_O_5_ heterostructure for advanced Li-Ion batteries. J. Alloys Compd..

[56] Shen F., Sun Z., He Q., Sun J., Kaner R.B., Shao Y. (2021). Niobium pentoxide based materials for high rate rechargeable electrochemical energy storage. Mater. Horiz..

[57] Nallapureddy R.R., Pallavolu M.R., Nallapureddy J., Yedluri A.K., Joo S.W. (2023). Z-scheme photocatalysis and photoelectrochemical platform with a Co_3_O_4_-CuO heterogeneous catalyst for the removal of water pollutants and generation of energy. J. Clean. Prod..

[58] Carvalho K.T.G., Nogueira A.E., Lopes O.F., Byzynski G., Ribeiro C. (2017). Synthesis of g-C_3_N_4_/Nb_2_O_5_ heterostructures and their application in the removal of organic pollutants under visible and ultraviolet irradiation. Ceram. Int..

[59] Hao J., Peng S., Li H., Dang S., Qin T., Wen Y., Huang J., Ma F., Gao D., Li F. (2018). A low crystallinity oxygen-vacancy-rich Co_3_O_4_ cathode for high-performance flexible asymmetric supercapacitors. J. Mater. Chem. A.

[60] Shi X., Quan S., Yang L., Liu C., Shi F. (2019). Anchoring Co_3_O_4_ on BiFeO_3_: Achieving high photocatalytic reduction in Cr(VI) and low cobalt leaching. J. Mater. Sci..

[61] Prabaharan D.D.M., Sadaiyandi K., Mahendran M., Sagadevan S. (2017). Precipitation method and characterization of cobalt oxide nanoparticles. Appl. Phys. A.

[62] Makula P., Pacia M., Macyk W. (2018). How to correctly determine the band gap energy of modified semiconductor photocatalysts based on UV-Vis spectra. J. Phys. Chem. Lett..

[63] Mohan H., Sethumathavan V., Fujii M., Ha G., Kim G., Lee H., Shin T. (2023). PdS Coupled Nb_2_O_5_ Superstructure for Photocatalytic Overall Water Splitting. ChemCatChem.

[64] Simon T., Carlson M.T., Stolarczyk J.K., Feldmann J. (2016). Electron transfer rate vs recombination losses in photocatalytic H_2_ generation on Pt-decorated CdS nanorods. ACS Energy Lett..

[65] He J., Hu Y., Wang Z., Lu W., Yang S., Wu G., Wang Y., Wang S., Gu H., Wang J. (2014). Hydrothermal growth and optical properties of Nb_2_O_5_ nanorod arrays. J. Mater. Chem. C.

[66] Gendo K.M., Feyisa Bogale R., Kenasa G. (2024). Green Synthesis, Characterization, and Evaluation of Photocatalytic and Antibacterial Activities of Co_3_O_4_-ZnO Nanocomposites Using *Calpurnia aurea* Leaf Extract. ACS Omega.

[67] Tahir N., Zahid M., Jillani A., Yaseen M., Abbas Q., Abdul shakoor R., Shahid I. (2023). Ternary silver tungstate-MoS_2_/graphene oxide heterostructure nanocomposite for enhanced photocatalysis under visible light and antibacterial activity. J. Photochem. Photobiol. A.

[68] Ray S.K., Dhakal D., Kshetri Y.K., Lee S.W. (2017). Cu-α-NiMoO_4_ photocatalyst for degradation of Methylene blue with pathways and antibacterial performance. J. Photochem. Photobiol. A.

[69] Munawar T., Nadeem M.S., Mukhtar F., Manzoor S., Ashiq N.M., Batool S., Hasan M., Iqbal F. (2023). Multifunctional dual Z-scheme heterostructured Sm_2_O_3_-WO_3_-La_2_O_3_ nanocomposite: Enhanced electrochemical, photocatalytic, and antibacterial properties. Adv. Powder Technol..

[70] Sapkota K.P., Lee I., Shrestha S., Islam A., Hanif A., Akter J., Hahn J.R. (2021). Coherent CuO-ZnO nanobullets maneuvered for photocatalytic hydrogen generation and degradation of a persistent water pollutant under visible-light illumination. J. Environ. Chem. Eng..

[71] Xu Y., Schoonen M.A.A. (2000). The absolute energy positions of conduction and valence bands of selected semiconducting minerals. Am. Mineral..

[72] Kumar K.V., Porkodi K., Rocha F. (2008). Langmuir–Hinshelwood kinetics—A theoretical study. Catal. Commun..

[73] Nassar M.Y., Aly H.M., Abdelrahman E.A., Moustafa M.E. (2017). Synthesis, characterization, and biological activity of some novel schiff bases and their Co(II) and Ni(II) complexes: A new route for Co_3_O_4_ and NiO nanoparticles for photocatalytic degradation of methylene blue dye. J. Mol. Struct..

[74] Yadav S., Yadav J., Kumar M., Saini K. (2022). Synthesis and characterization of nickel oxide/cobalt oxide nanocomposite for effective degradation of methylene blue and their comparative electrochemical study as electrode material for supercapacitor application. Int. J. Hydrogen Energy.

[75] Saranya J., Ranjith K.S., Saravanan P., Mangalaraj D., Kumar R.T.R. (2014). Cobalt-doped cerium oxide nanoparticles: Enhanced photocatalytic activity under UV and visible light irradiation. Mater. Sci. Semicond. Process..

[76] Chinnathambi A., Nasif O., Alharbi S.A., Khan S.S. (2021). Enhanced optoelectronic properties of multifunctional MnFe_2_O_4_ nanorods decorated Co_3_O_4_ nanoheterostructure: Photocatalytic activity and antibacterial behavior. Mater. Sci. Semicond. Process..

[77] Zarrin S., Heshmatpour F. (2018). Photocatalytic activity of TiO_2_/Nb_2_O_5_/PANI and TiO_2_/Nb_2_O_5_/RGO as new nanocomposites for degradation of organic pollutants. J. Hazard. Mater..

[78] Oliveira L.C., Oliveira H.S., Mayrink G., Mansur H.S., Mansur A.A., Moreira R.L. (2014). One-pot synthesis of CdS@ Nb_2_O_5_ core–shell nanostructures with enhanced photocatalytic activity. Appl. Catal. B.

[79] Moraes N.P.d., Silva M.L.C.P.d., Rodrigues L.A. (2018). Effect of metal doping in the photocatalytic properties of carbon xerogel-Nb_2_O_5_ composite towards visible light degradation of methylene blue. Mater. Lett..

[80] Danish M., Pandey A. (2016). Influence of thickness and calcination under ammonia gas flow on topographical, optical and photocatalytic properties of Nb_2_O_5_ thin films prepared by sol–gel: A comparative study. J. Mater. Sci. Mater. Electron..

[81] Ajami A., Sheibani S., Ataie A. (2024). S-scheme CoFe_2_O_4_/g-C_3_N_4_ nanocomposite with high photocatalytic activity and antibacterial capability under visible light irradiation. J. Mater. Res. Technol..

[82] Zhou B., Zhao X., Liu H., Qu J., Huang C.P. (2010). Visible-light sensitive cobalt-doped BiVO_4_ (Co-BiVO_4_) photocatalytic composites for the degradation of methylene blue dye in dilute aqueous solutions. Appl. Catal. B.

[83] Brites-Nóbrega F.F., Lacerda I.A., Santos S.V., Amorim C.C., Santana V.S., Fernandes-Machado N.R., Ardisson J.D., Henriques A.B., Leão M.M. (2015). Synthesis and characterization of new NaX zeolite-supported Nb, Zn, and Fe photocatalysts activated by visible radiation for application in wastewater treatment. Catal. Today.

[84] Mobeen A.A., Jasmine S.S.K., Sundaram R., Maria M.C., Kaviyarasu K., Letsholathebe D., Mohamed S.B., Kennedy J., Maaza M. (2018). Antibacterial, magnetic, optical and humidity sensor studies of beta-CoMoO_4_–Co_3_O_4_ nanocomposites and its synthesis and characterization. J. Photochem. Photobiol. B.

