# Supplementary material for: Visible-Light-Driven Co3O4/Nb2O5 Heterojunction Nanocomposites for Efficient Photocatalytic and Antimicrobial Performance in Wastewater Treatment
Source: Molecules. 2025 Jun 12;30(12):2561. doi: 10.3390/molecules30122561 (PMC12196055; doi:10.3390/molecules30122561)
Supplement: Supplementary file 1 [file molecules-30-02561-s001.zip › molecules-3646126-supplementary.pdf]

Visible-Light-Driven Co_3O_4/Nb_2O_5 Heterojunction Nanocomposites for Efficient Photocatalytic and Antimicrobial Performance in Wastewater Treatment

Anil Pandey [1,†], Santu Shrestha [1,†], Rupesh Kandel [2], Narayan Gyawali [1], Subas Acharya [1], Pujan Nepal [1], Binod Gaire [1], Vince Fualo [1] and Jae Ryang Hahn [1,3,*]

[1] Department of Chemistry, Jeonbuk National University, Jeonju 54896, Republic of Korea; pandeyan2001@gmail.com (A.P.); santu_shrestha@hotmail.com (S.S.); naran.gywli@gmail.com (N.G.); link2subas@gmail.com (S.A.); pujannepal25@gmail.com (P.N.); gairebinod@gmail.com (B.G.); fualo.vc@gmail.com (V.F.)
[2] Department of Bionanotechnology and Bioconvergence Engineering, Graduate School, Jeonbuk National University, Jeonju 54896, Republic of Korea; rupkandel87@gmail.com
[3] Textile Engineering, Chemistry and Science, North Carolina State University, 2401 Research Dr., Raleigh, NC 27695-8301, USA
* Correspondence: jhahn@ncsu.edu or jrhahn@jbnu.ac.kr
† These authors contributed equally to this work

.

Characterization techniques

Field Emission Scanning Electron Microscope (FE-SEM, SU-8230, Hitachi, Japan) and High-Resolution Transmission Electron Microscope (HR-TEM, JEM-2200FS, JEOL, Japan) were used to examine the morphological characteristics of the nanocomposites. During FE-SEM investigations, energy-dispersive X-ray spectroscopy (EDX, SU-8230 Hitachi, Japan) was employed to ascertain the purity and elemental composition of the nanocomposite. X-ray Diffraction (XRD) analysis was conducted with a scan rate of 5° min^{-1} in the range of 6-90° using Cu Kα as the X-ray source (λ = 0.154 nm) using X-ray diffractometer (Smart Lab, Rigaku, Japan). Fourier transform infrared (FTIR) spectroscopy (Nicolet iS5, Thermo Fischer Scientific, Waltham, MA, USA) was applied to investigate the functional groups of the nanocomposites. UV-Vis spectrophotometry (Lambda 25, PerkinElmer, Waltham, MA, USA) was used to analyze the spectroscopic properties of the nanocomposites. High-performance XPS (Al Kα/1486.6 eV/Nexa XPS system, Thermo Scientific, Waltham, MA, USA) was used to study the chemical state of the elements. Photoluminescence (PL) properties in the hetero nanocomposites were investigated using a fluorometer (LS55, PerkinElmer, Waltham, MA, USA). The Liquid Chromatography-Mass Spectroscopy (6410 Triple Quad, Agilent Technologies, Santa Clara, CA, USA) was employed to detect degradation fragments of MB. Electrochemical impedance measurement was carried out with an electrochemical workstation (ZIVE SP1, WonATech, South Korea) equipped with a standard three-electrode system. An Ag/AgCl electrode (saturated with KCl) as a reference electrode (Basi, USA) and a carbon rod as a counter electrode were used in 0.2 M Na_2SO_4 electrolyte solution. Test sample ink made using 2 mg of catalyst was drop-cast onto a glassy carbon electrode to obtain the working electrode.

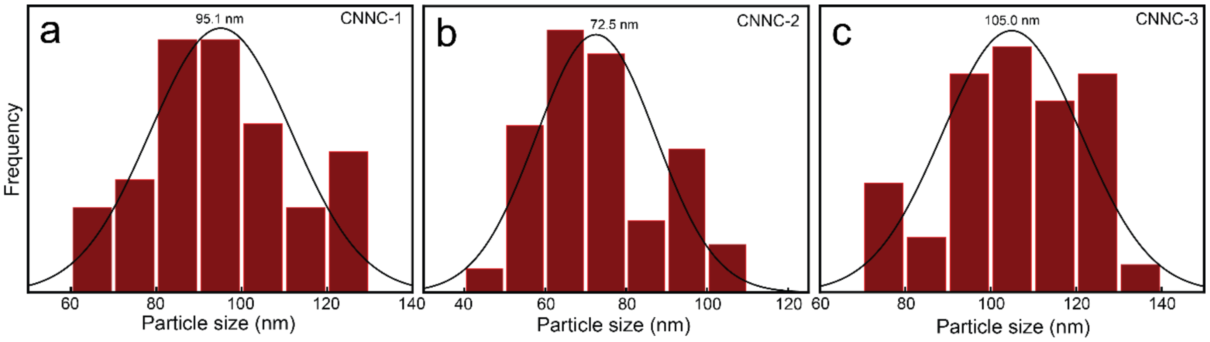

Figure S1. Particle size distribution plot for (a) CNNC-1, (b) CNNC-2, and (c) CNNC-3.

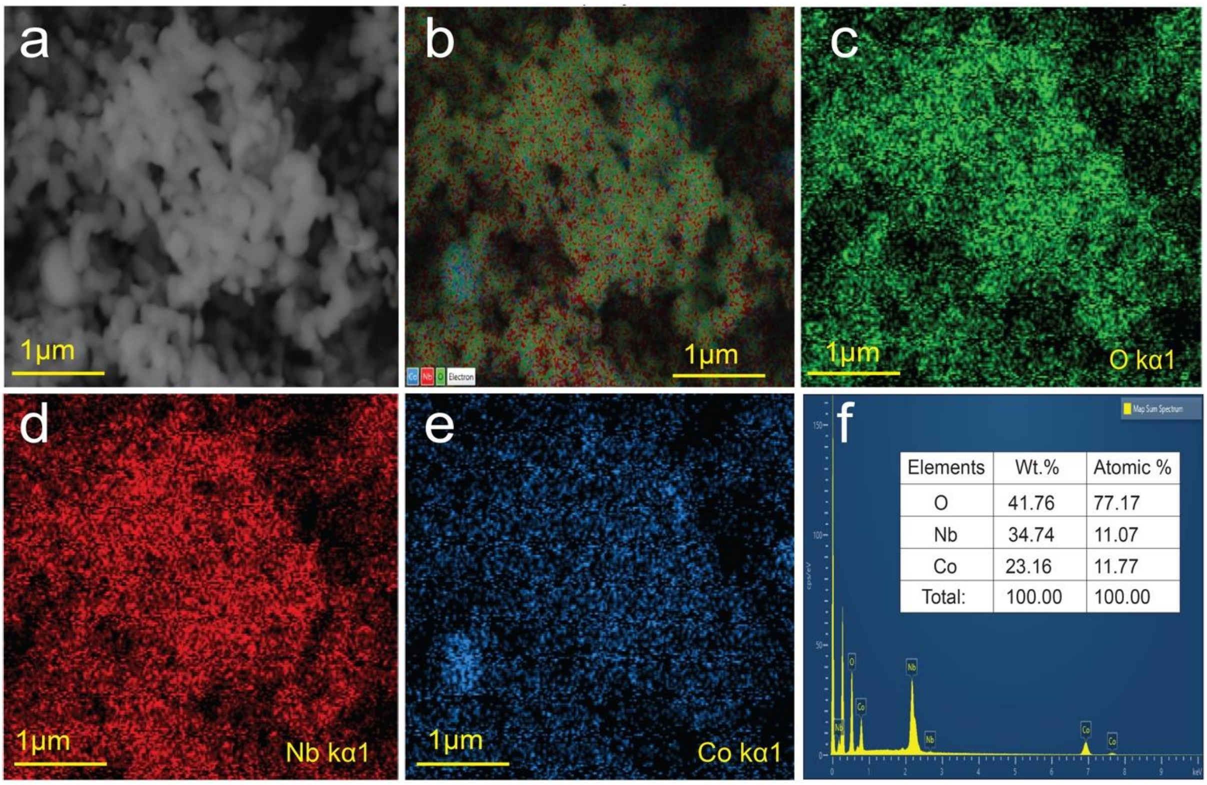

Figure S2. (a) FE-SEM image, (b) EDS layered image, (c-e) elemental distribution (O, Nb, and Co), and (f) EDS spectrum of CNNC-1.

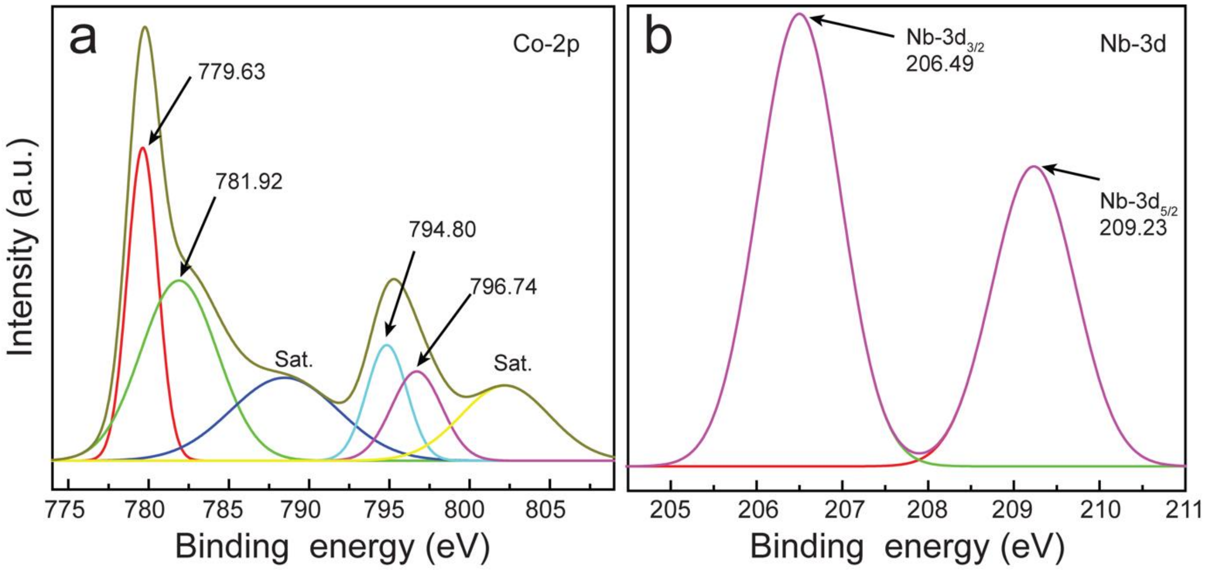

Figure S3. XPS spectra: (a) Co-2*p* in individual CONP and (b) Nb-3*d* in individual NONP.

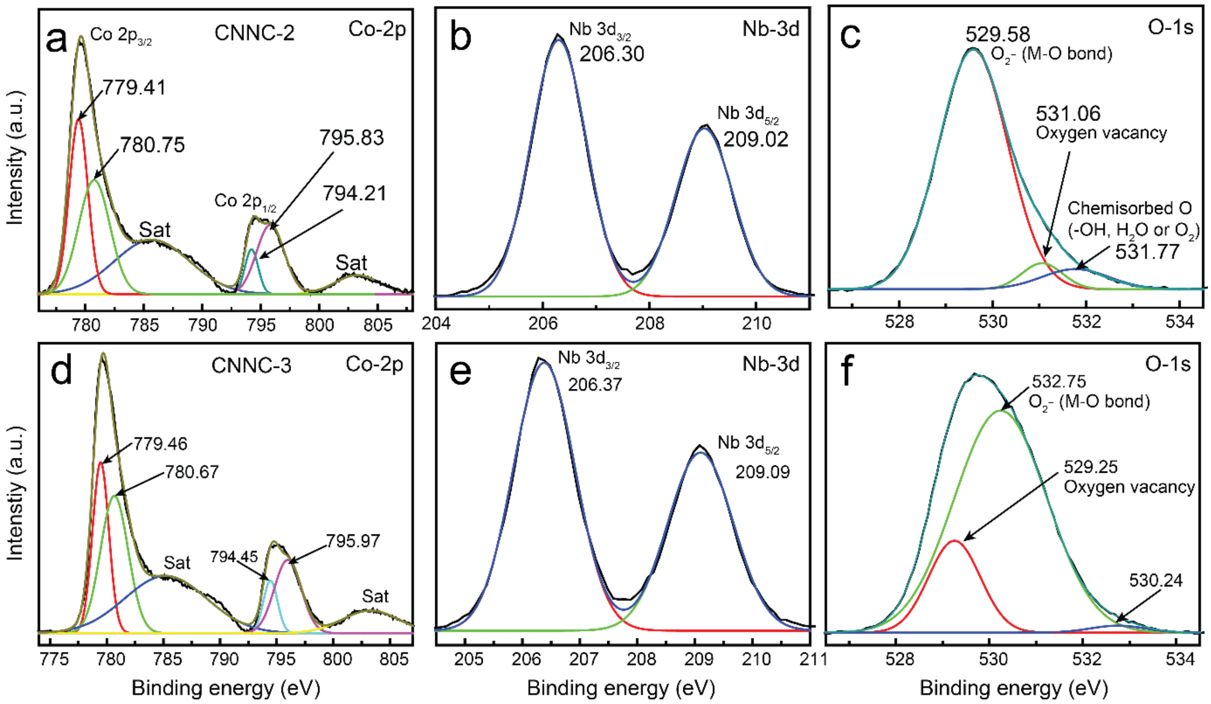

Figure S4. XPS spectra: Co-2*p*, Nb-3*d*, and O-1s in (a-c) CNNC-2 and (d-f) CNNC-3.

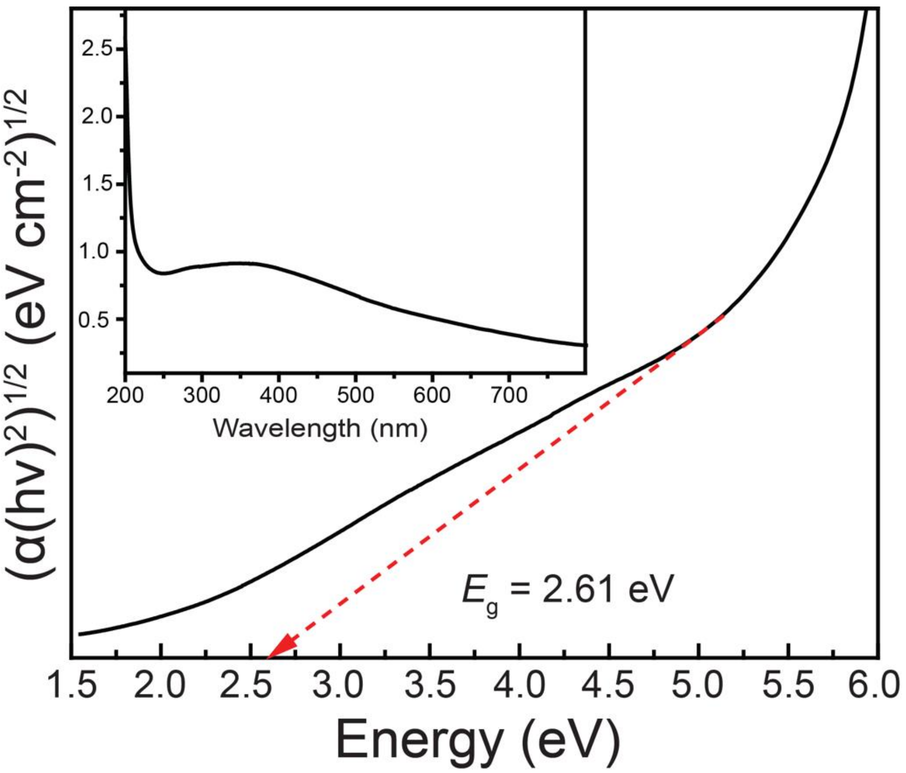

Figure S5. Tauc plot of CNNC-3.

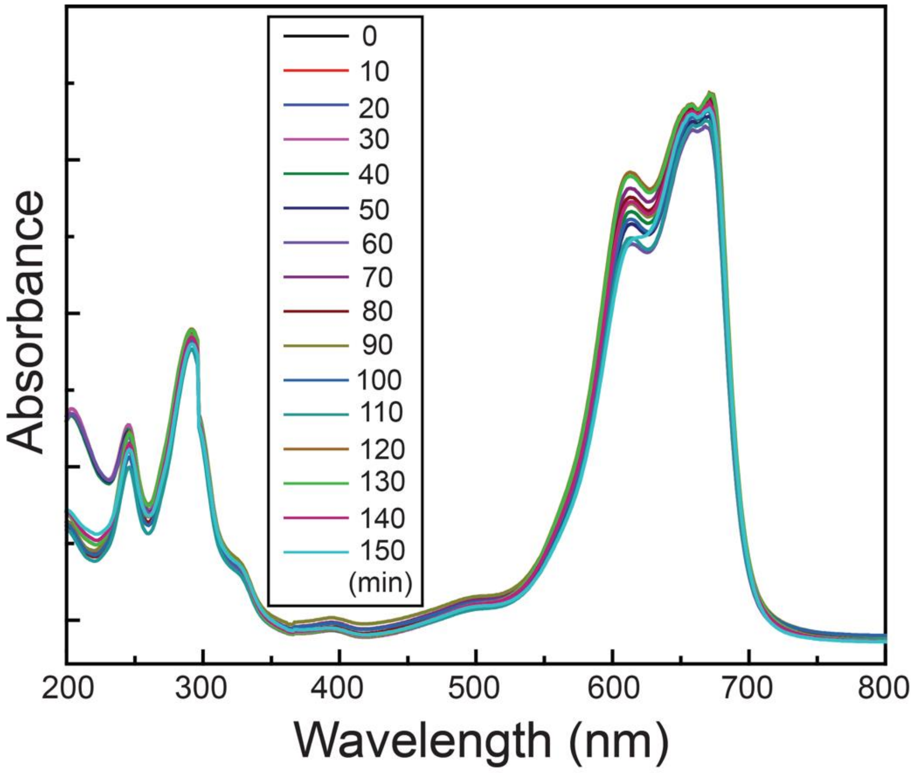

Figure S6. Degradation spectra of MB in the absence of a photocatalyst.

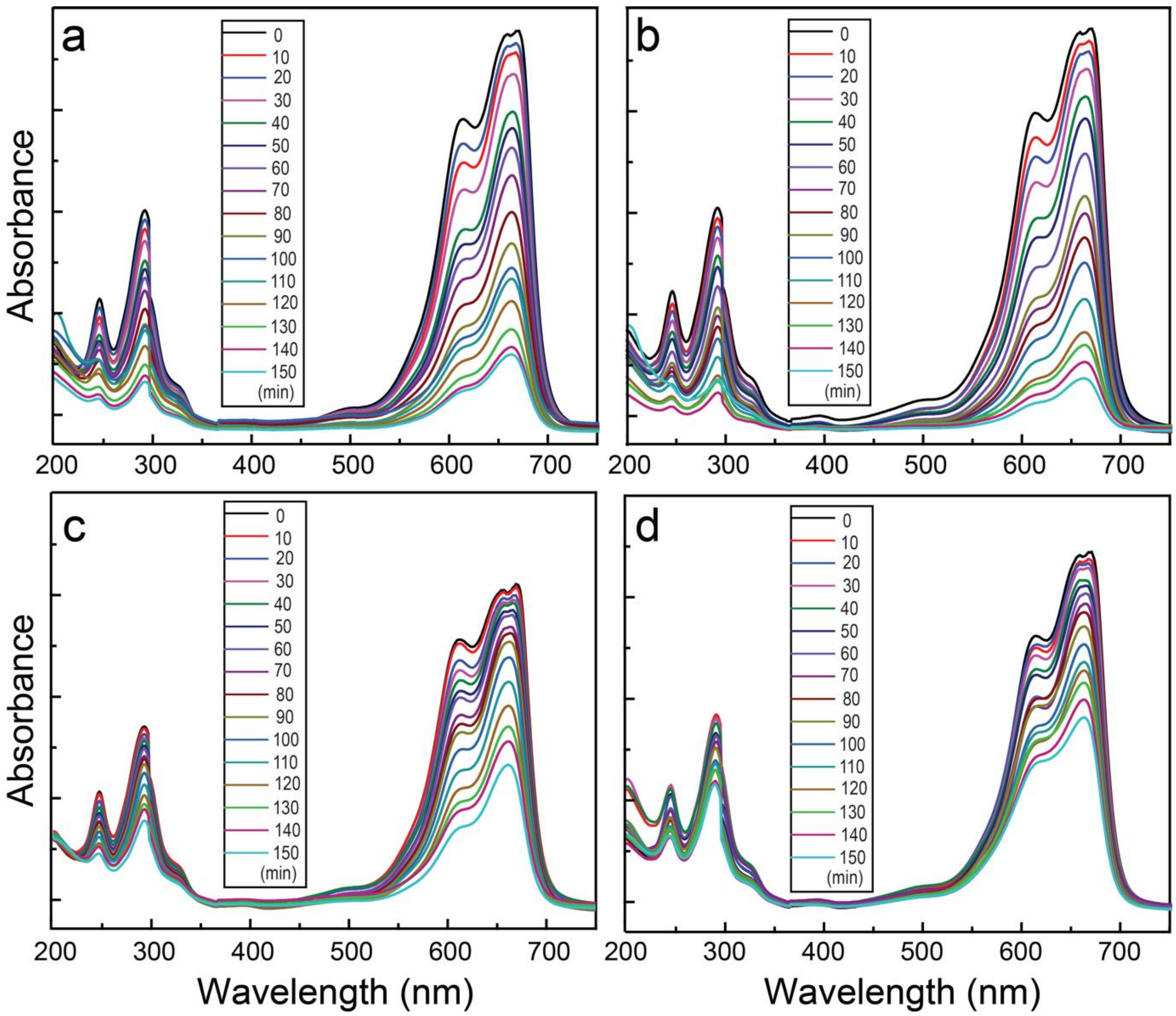

Figure S7. UV-Vis spectra of MB degradation for (a) CNNC-2, (b) CNNC-3, (c) CONP, and (d) NONP.

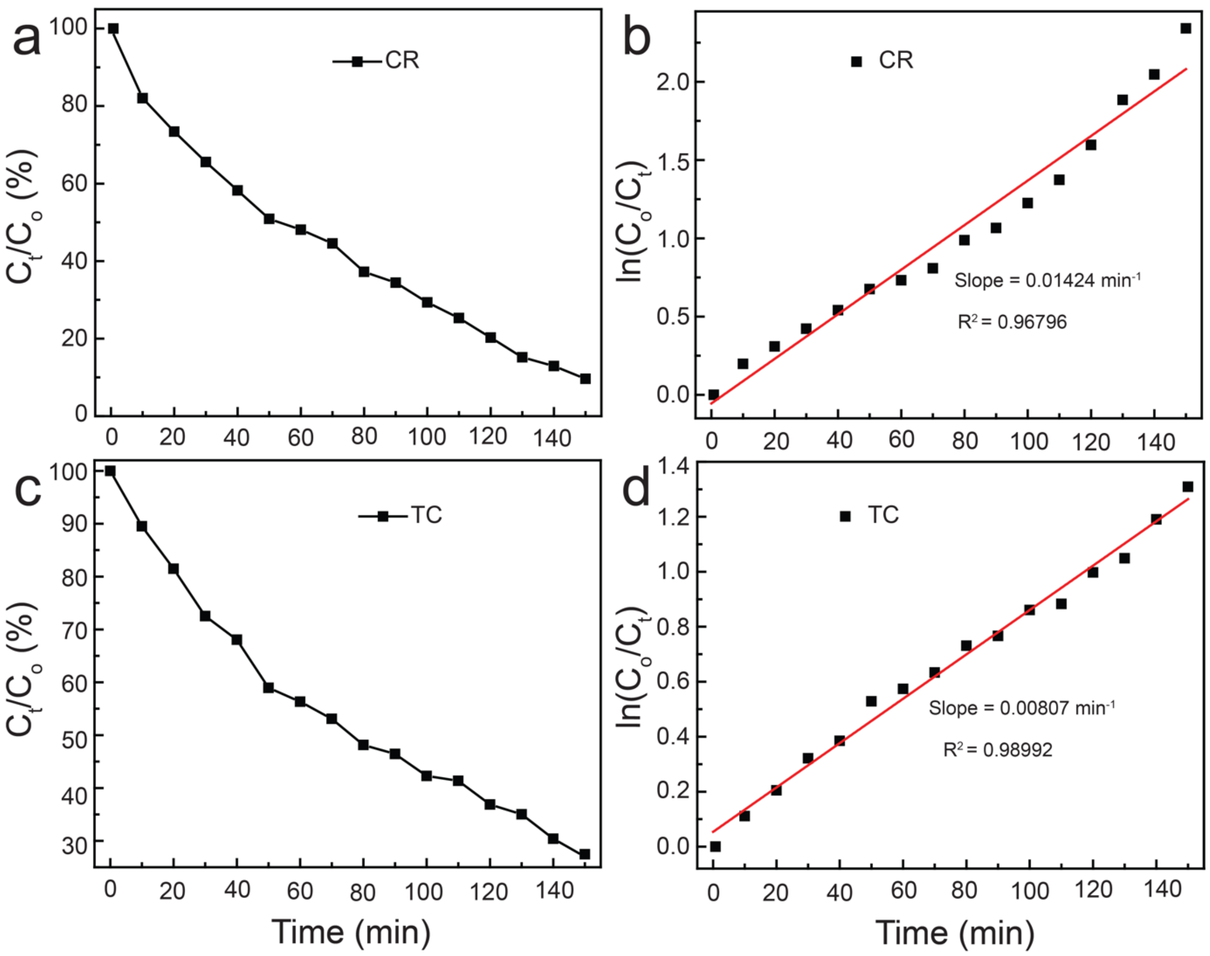

Figure S8. Degradation plot and kinetic plot of CNNC-1 for (a, b) CR and (c, d) TC.

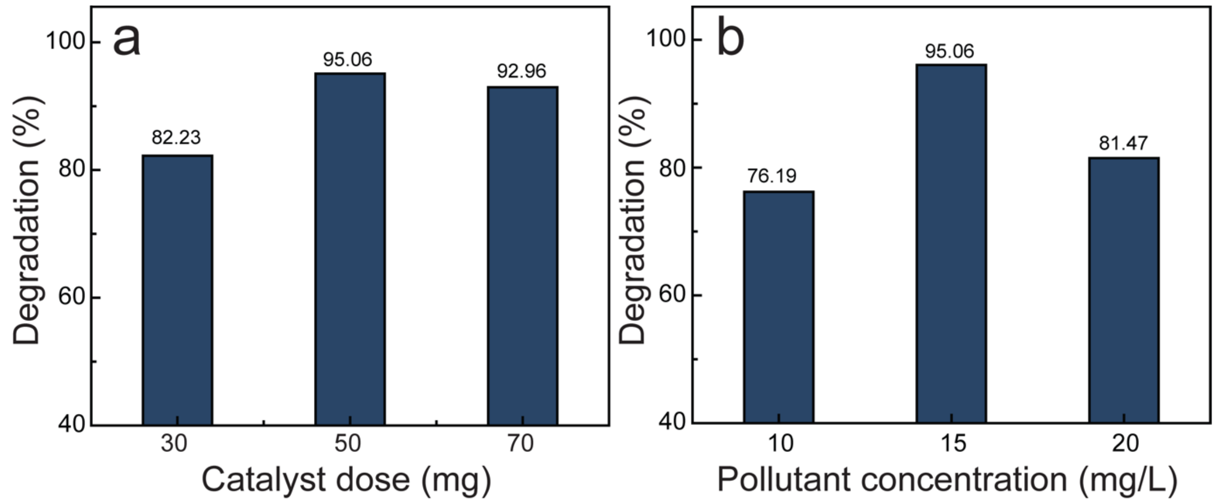

Figure S9. MB degradation bar diagram of CNNC-1 for the effect of (a) catalyst dose and (b) concentration.

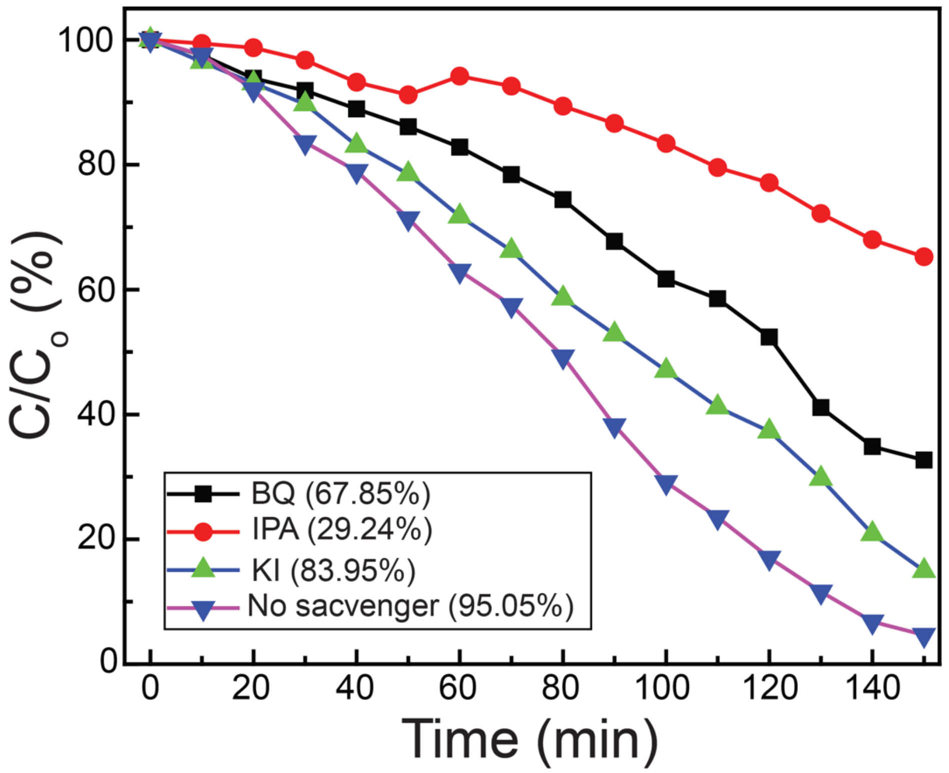

Figure S10. Effect of charge carrier scavengers for MB degradation using CNNC-1.

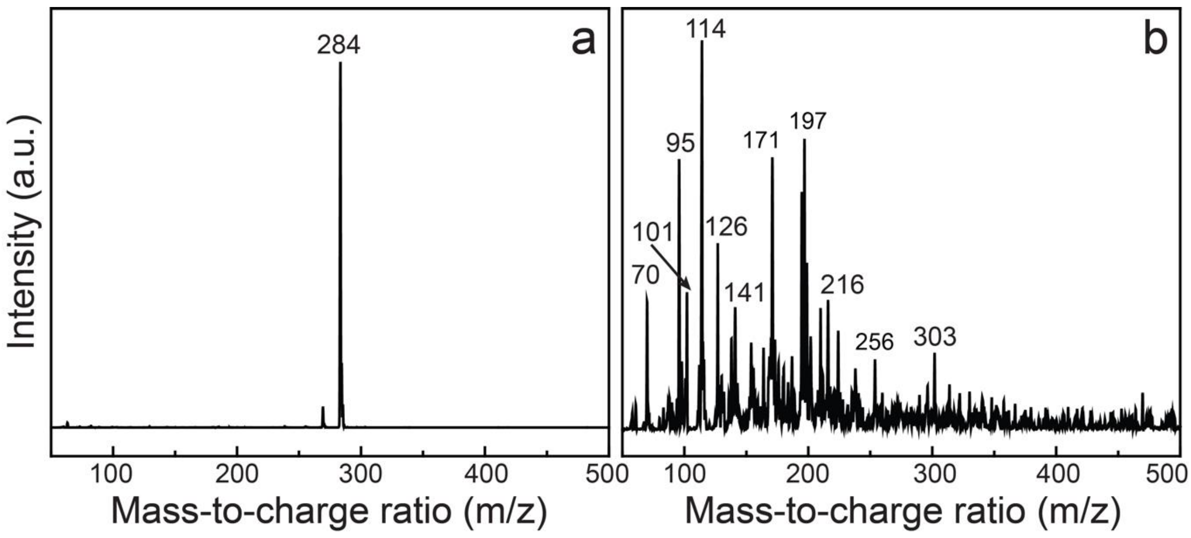

Figure S11. LC-MS spectra of (a) fresh MB and (b) degraded MB using CNNC-1.

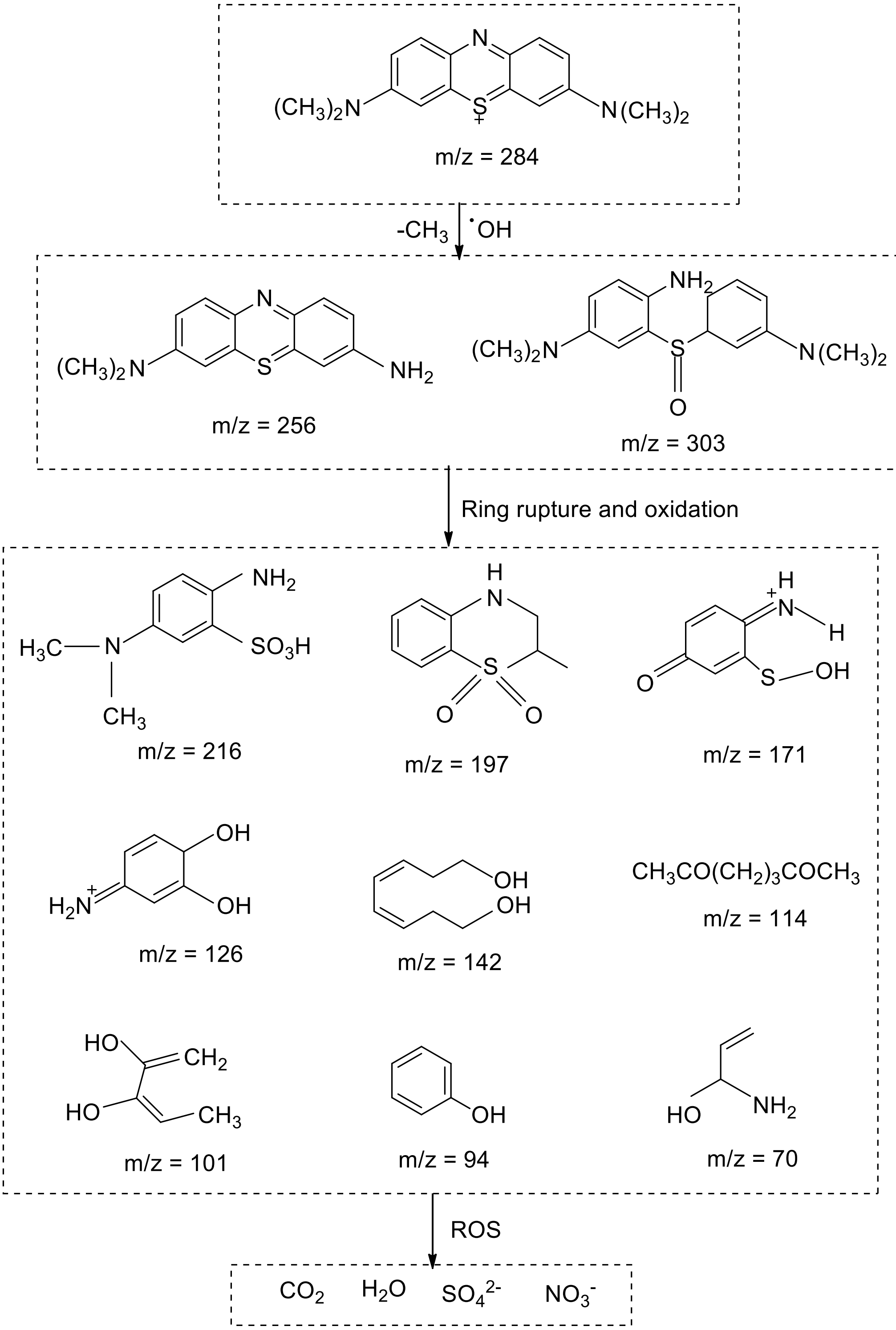

Figure S12. Proposed degradation pathway of MB.

Table S1. The average particle size (nm) of the Nanocomposite

Nanocomposite	Average particle size (nm)
CNNC-1	95.1
CNNC-2	72.5
CNNC-3	105.0

Table S2. Comparison of the BET surface area and pore volume (BJH) of samples.

**Sample**	**Surface area (m^2/g)**	**Pore volume (cm^3/g)**
NONP	0.7979	0.001928
CONP	3.0613	0.017448
CNNC-1	14.3093	0.039245

Table S3. The structural parameters of synthesized materials

**Parameters**	**CONP**	**NONP**	**CNNC-1**	**CNNC-2**	**CNNC-3**
Crystallinity (%)	30.28	61.17	42.86	31.7	49.97
Dislocation density (nm^{-2})	0.0019	0.0017	0.0016	0.0011	0.0019
Lattice constant	a = b = c = 8.12	a = 6.19 c = 3.94 b = 28.73	-	-	-
Crystallite size (W-H-)	-	-	24.61	29.02	22.52
Crystallite size (Scherrer equation)	-	-	17.45	19.05	20.68

Table S4. R^2 value of the pseudo-first-order reaction of the prepared materials

Catalyst	Blank	NONP	CONP	CNNC-1 (1:1)	CNNC-2 (1:2)	CNNC-3 (2:1)
R^2	0.99308	0.86246	0.94289	0.88932	0.95528	0.93957

Table S5. Comparison of the photocatalytic efficiency of Co_3O_4/Nb_2O_5 composites and related composite materials.

Composite	Synthesis method	Morphology	Pollutant	Pollutant concentration	Composite dose	Degradation (%)	Light source/degradation time	Ref.
**Co_3O_4 NPs**	Thermal decomposition	Spherical Irregular	MB	10 ppm	0.1g	55.71	250W xenon arc lamp/420 min	[1]
**NiO/Co_3O_4**	Wet chemical	Sheet shaped	MB	10 ppm	100 mg	89.88	Sunlight/360 min	[2]
**Co-doped/CeO_2**	Coprecipitaion	Irregular shape	MB	15 ppm	0.1g	89	Visible light/420 min	[3]
**$MnFe_2O_4$-Co_3O_4**	Sonochemical	Nanorods clusters	MB	25 mg/L	150 mg	80	Visible light/200 min	[4]
**TiO_2/Nb_2O_5/RGO**	Hydrothermal	Spherical cluster	MB	20 mg/dm3	$1.2x10^{-4}$ mmol	97	300 W Xe lamp /240 min	[5]
**$CdS@Nb_2O_5$**	Precipitation	Core-shell	MB	50 mg/L	60mg	80	125 W Hg lamp/180 min	[6]
**Carbon xerogel-Nb_2O_5**	Precipitation	Irregular shape	MB	10 mg/L	0.5g/L	30	Visible light/300 min	[7]
**Nb_2O_5**	Sol-gel	Thin film	MB	4 mg/L	-	84.13	300 W Hg lamp/480 min	[8]
**$CoFe_2O_4$/g-C_3N_4**	Co-precipitation	Decorated nanosheet	MB	10 mg/L	0.02g	97.3	Visible light/180 min	[9]
**Co-$BiVO_4$**	Heteronuclear complexing	irregular	MB	10 mg/L	0.1g	85	Visible light/5h	[10]
**Nb_2O_5/NaX zeolite**	Wet impregnation	Bulky	MB	20 mg/L	5g/L	60	80 W Xe lamp/300 min	[11]
**Co_3O_4/Nb_2O_5**	Co-crystallization/ Annealing	Porous/orthorhombic	MB<br>CR<br>TC	15 ppm	50 mg	95.05<br>92.37<br>72.56	Visible light/150 min	**Our work**

Table S6. Comparison of the antibacterial activity of Co_3O_4/Nb_2O_5 composites and relevant composite materials.

**Catalyst name**	**Catalyst dose (mg/mL)**	**Activity to *E. coli* growth reduction (mm)/plate count (%)**	**Activity to *S. aureus* growth reduction**	**Reference**
CNNC	5	6.8 mm	9.3	Our work
Co_3O_4–ZnO	100	7.5 mm	8.5 mm	[12]
Co/Co_3O_4	NA	8.1	0 mm	[13]
CaF_2-CaO-B_2O_3-P_2O_5-SrOglass/Nb_2O_5	NA	30	25	[14]
Hap-Nb_2O_5	NA	19%	20%	[15]
β-$CoMoO_4$-Co_3O_4	1.56	12 mm	14 mm	[16]
Graphene – CoO_x/MnO_x	20	13 mm	14 mm	[17]

References:

1. Nassar, M.Y.; Aly, H.M.; Abdelrahman, E.A.; Moustafa, M.E. Synthesis, characterization, and biological activity of some novel schiff bases and their Co(II) and Ni(II) complexes: a new route for Co_3O_4 and NiO nanoparticles for photocatalytic degradation of methylene blue dye. *J. Mol. Struct.* **2017**, *1143*, 462-471. https://doi.org/10.1016/j.molstruc.2017.04.118
2. Yadav, S.; Yadav, J.; Kumar, M.; Saini, K. Synthesis and characterization of nickel oxide/cobalt oxide nanocomposite for effective degradation of methylene blue and their comparative electrochemical study as electrode material for supercapacitor application. *Int. J. Hydrogen Energy* **2022**, *47*, 41684-41697. https://doi.org/10.1016/j.ijhydene.2022.02.011
3. Saranya, J.; Ranjith, K.S.; Saravanan, P.; Mangalaraj, D.; Kumar, R.T.R. Cobalt-doped cerium oxide nanoparticles: enhanced photocatalytic activity under UV and visible light irradiation. *Mater. sci. semicond. process.* **2014**, *26*, 218-224. https://doi.org/10.1016/j.mssp.2014.03.054
4. Chinnathambi, A.; Nasif, O.; Alharbi, S.A.; Khan, S.S. Enhanced optoelectronic properties of multifunctional $MnFe_2O_4$ nanorods decorated Co_3O_4 nanoheterostructure: photocatalytic activity and antibacterial behavior. *Mater. Sci. Semicond. Process.* **2021**, *134*, 105992. https://doi.org/10.1016/j.mssp.2021.105992
5. Zarrin, S.; Heshmatpour, F. Photocatalytic activity of TiO_2/Nb_2O_5/PANI and TiO_2/Nb_2O_5/RGO as new nanocomposites for degradation of organic pollutants. *J. hazard. mater.* **2018**, *351*, 147-159. https://doi.org/10.1016/j.jhazmat.2018.02.052
6. Oliveira, L.C.; Oliveira, H.S.; Mayrink, G.; Mansur, H.S.; Mansur, A.A.; Moreira, R.L. One-pot synthesis of CdS@ Nb_2O_5 core–shell nanostructures with enhanced photocatalytic activity. *Appl. Catal., B* **2014**, *152*, 403-412. https://doi.org/10.1016/j.apcatb.2014.01.025
7. Moraes, N.P.d.; Silva, M.L.C.P.d.; Rodrigues, L.A. Effect of metal doping in the photocatalytic properties of carbon xerogel-Nb_2O_5 composite towards visible light degradation of methylene blue. *Mater. Lett.* **2018**, *228*, 486-489. https://doi.org/10.1016/j.matlet.2018.06.095.
8. Danish, M.; Pandey, A. Influence of thickness and calcination under ammonia gas flow on topographical, optical and photocatalytic properties of Nb_2O_5 thin films prepared by sol–gel: a comparative study. *J. Mater. Sci.: Mater. Electron.* **2016**, *27*, 6939-6946. https://doi.org/10.1007/s10854-016-4648-5
9. Ajami, A.; Sheibani, S.; Ataie, A. S-scheme $CoFe_2O_4$/g-C_3N_4 nanocomposite with high photocatalytic activity and antibacterial capability under visible light irradiation. *J. Mater. Res. Technol.* **2024**, *30*, 2168-2185. https://doi.org/10.1016/j.jmrt.2024.04.010
10. Zhou, B.; Zhao, X.; Liu, H.; Qu, J.; Huang, C.P. Visible-light sensitive cobalt-doped $BiVO_4$ (Co-$BiVO_4$) photocatalytic composites for the degradation of methylene blue dye in dilute aqueous solutions. *Appl. Catal. B.*, **2010**, *99*, 214-221. https://doi.org/10.1016/j.apcatb.2010.06.022.
11. Brites-Nóbrega, F.F.; Lacerda, I.A.; Santos, S.V.; Amorim, C.C.; Santana, V.S.; Fernandes-Machado, N.R.; Ardisson, J.D.; Henriques, A.B.; Leão, M.M. Synthesis and characterization of new NaX zeolite-supported Nb, Zn, and Fe photocatalysts activated by visible radiation for application in wastewater treatment. *Catal. Today* **2015**, *240*, 168-175. https://doi.org/10.1016/j.cattod.2014.06.036.
12. Gendo, K.M.; Feyisa B.R.; Kenasa, G. Green Synthesis, Characterization, and Evaluation of Photocatalytic and Antibacterial Activities of Co_3O_4-ZnO Nanocomposites Using Calpurnia aurea Leaf Extract. *ACS Omega* **2024**, *9*, 28354-28371. https://doi.org/10.1021/acsomega.4c01595.
13. Yousefi, S.R.; Alshamsi, H.A.; Amiri, O.; Salavati-Niasari, M. Synthesis, characterization and application of Co/Co_3O_4 nanocomposites as an effective photocatalyst for discoloration of organic dye contaminants in wastewater and antibacterial properties. *J. Mol. Liq.* **2021**, *337*. https://doi.org/10.1016/j.molliq.2021.116405.
14. Madhavi, B.; Siva S.R.A.; Syam P.P.; Prasad, A.; Pavani K.D.P.; Ravi K.V.; Veeraiah, N. The impact of Nb_2O_5 on in-vitro bioactivity and antibacterial activity of CaF_2–CaO–B_2O_3–P_2O_5–SrO glass system. *Ceram. Int.* **2021**, *47*, 28328-28337. https://doi.org/10.1016/j.ceramint.2021.06.250.

15. Safavi, M.S.; Khalil-Allafi, J.; Restivo, E.; Ghalandarzadeh, A.; Hosseini, M.; Dacarro, G.; Malavasi, L.; Milella, A.; Listorti, A.; Visai, L. Enhanced in vitro immersion behavior and antibacterial activity of NiTi orthopedic biomaterial by HAp-Nb_2O_5 composite deposits. *Sci. Rep.* **2023**, *13*, 16045. https://doi.org/10.1038/s41598-023-43393-3.
16. Mobeen A. A.; Jasmine S.S.K.; Sundaram, R.; Maria M.C.; Kaviyarasu, K.; Letsholathebe, D.; Mohamed, S.B.; Kennedy, J.; Maaza, M. Antibacterial, magnetic, optical and humidity sensor studies of beta-$CoMoO_4$ - Co_3O_4 nanocomposites and its synthesis and characterization. *J. Photochem. Photobiol., B* **2018**, *183*, 233-241. https://doi.org/10.1016/j.jphotobiol.2018.04.034.
17. Liyanaarachchi, H.; Thambiliyagodage, C.; Jayanetti, M.; Ekanayake, G.; Wijayawardana, S.; Samarakoon, U. The photocatalytic and antibacterial activity of graphene oxide coupled CoO_x /MnO_x nanocomposites. *Environ. Technol. Innovation* **2025**, *37*. https://doi.org/10.1016/j.eti.2024.103984.
